# Deletion of a YEDL sequence in SLC35D3 perturbs its targeting to platelet dense granule precursors

**DOI:** 10.1016/j.jbc.2026.113335

**Published:** 2026-07-16

**Authors:** Chentong Wang, Yuanying Chen, Wenyan Zhang, Yefeng Yuan, Qiaochu Wang, Chanjuan Hao, Wei Li

**Affiliations:** 1 Laboratory for Genetics of Birth Defects, Beijing Pediatric Research Institute, MOE Key Laboratory of Major Diseases in Children, Genetics and Birth Defects Control Center, National Center for Children’s Health, Beijing Children’s Hospital, Capital Medical University, Beijing, China; 2 Henan Key Laboratory of Genetic and Developmental Disorders, Institute of Children’s Health, Henan Academy of Medical Sciences, Zhengzhou, China; 3 Zhengzhou Hospital of Beijing Children’s Hospital, Zhengzhou, China

**Keywords:** protein trafficking, adaptor protein complex, BLOC-1, SLC35D3, dense granules

## Abstract

Solute carrier family 35 member D3 (SLC35D3) is an integral membrane protein required for platelet dense granule (DG) biogenesis. SLC35D3-null mice display defective DG formation and prolonged bleeding time, yet the underlying mechanism remains unclear. Proper targeting of SLC35D3 to DGs is crucial for their physiological function. Here, we identified the Y^311^EDL sequence located in the C-terminal tail of SLC35D3, as a key determinant for its sorting into DG precursors. Deletion of this motif resulted in the accumulation of SLC35D3 on the plasma membrane. Using immunoprecipitation and pulldown assays, we demonstrated that the Y^311^EDL deletion also impaired SLC35D3 binding to the adaptor protein complex-3 (AP-3), although substitution of Y^311^EDL with alanines did not disrupt sorting or AP-3 binding. Moreover, we found that the interaction between SLC35D3 and the biogenesis of lysosome-related organelles complex 1 (BLOC-1) was dependent on AP-3 for endolysosomal targeting. We also showed that AP-3 and BLOC-1 deficiencies led to the lysosomal degradation of SLC35D3. This likely explains the observed reduction in SLC35D3 levels in platelets from AP-3 or BLOC-1 deficient mice. Together, our findings suggest that the Y^311^EDL sequence is required for the proper intracellular sorting of SLC35D3 to platelet DG precursors, thereby ensuring their functions.

Dense granule (DG) is a type of lysosome-related organelles (LROs) specifically present in platelets. DG deficiency results in bleeding tendency, commonly observed in Hermansky-Pudlak syndrome (HPS) and Chediak-Higashi syndrome (CHS) (1). SLC35D3 is a nucleotide sugar transporter localized to synaptic vesicles (2). Its deficiency causes DG defects in mice (3), highlighting the significance of SLC35D3 in platelet DG biogenesis (4). However, the molecular mechanisms underlying the sorting of SLC35D3 into platelet DGs remain unclear.

Intracellular proteins must be accurately targeted to specific compartments of the endomembrane system, such as endosomes, lysosomes, and LROs, to execute their biological functions (5). A fundamental prerequisite for understanding the trafficking routes of these proteins to their target organelles is the identification and characterization of protein sorting signals. Key regulators in this process include several sorting complexes, notably adaptor protein complexes (APs) and coat protein complexes (COPs) (6). To date, five distinct AP complexes (AP-1 to AP-5) have been identified, each localized to different intracellular compartments and mediating membrane trafficking through specific pathways (7, 8, 9, 10). AP complexes mediate cargo sorting through the precise recognition of sorting signals, which are primarily located within the cytosolic domains of integral membrane proteins. These sorting signals are typically presented as short linear motifs, which are predominantly recognized as tyrosine-based signals motif (YXXØ, where Ø represents a bulky hydrophobic amino acid) and di-leucine-based signals motif ([DE]XXXL[LI]) (6). Reduced levels of SLC35D3 were found in AP-3 and biogenesis of lysosome-related organelles complex 1 (BLOC-1)-deficient mouse platelets, indicating that mis-sorting of SLC35D3 may lead to its destabilization or alter its intracellular trafficking routes (4). However, how AP-3 and BLOC-1 are involved in SLC35D3 trafficking is unknown.

HPS arises from mutations in components that orchestrate the formation and trafficking of LROs. These genetic defects cause multi-LRO dysfunction, affecting melanosomes, DGs, pulmonary lamellar bodies (LBs), and endothelial Weibel-Palade bodies (WPBs) (11). In mammals, LROs are derived from endo-lysosomes through shared biogenesis pathways (12). It has been shown that SLC35D3 delivery from megakaryocyte early endosomes is critical for DG biogenesis and is differentially defective in Hermansky-Pudlak syndrome models (4). Similarly, TMEM163 has been implicated in DG biogenesis (13) and also localizes to SVs (14). We recently identified a conserved N-terminal acidic dileucine motif (L^69^L^70^) in TMEM163 that is essential for interactions with AP-3 and BLOC-1 sequentially and competitively for its targeting to DGs (15). However, each specific cargo of LROs may undergo a unique sorting machinery that needs to be specified.

In this study, a putative sorting signal, Y^311^EDL, located in the cytosolic domain of SLC35D3, is important for its association with AP-3, rather than AP-1 or AP-2, thereby being involved in the SLC35D3 intracellular trafficking. Moreover, the association between SLC35D3 and the BLOC-1 depends on the presence of AP-3. Deletion of the Y^311^EDL sequence in SLC35D3 disrupts its proper targeting to platelet DG precursors, resulting in mislocalization to the plasma membrane (PM). Additionally, loss of AP-3 or BLOC-1 reduces the stability of SLC35D3, promoting its degradation *via* the lysosomal degradation pathway. These data suggest that SLC35D3 targets platelet DG precursors through an AP-3/BLOC-1-dependent mechanism, providing a molecular basis for understanding DG biogenesis mediated by SLC35D3.

## Results

### Identification of the sorting signals in SLC35D3

We predicted that SLC35D3 possessed two potential tyrosine-based signals (Y^311^EDL and Y^389^LEV) and three potential di-leucine-based motifs (RRLGL^64^I, RGVPL^372^V, and EVWRL^395^V), which may interact with the AP complexes using the ELM server (16) (Fig. S1*A*). The di-leucine-based motif located at amino acids 60 to 65 resides within the predicted transmembrane domain, thereby precluding its role as a sorting signal for protein trafficking in the endocytic pathway (Fig. S1*B*) (6). For the remaining di-leucine-based sorting signals, one motif RGVPL^372^V was not conserved among most species, which excluded it as a high-ranking signal (Fig. S1*C*).

**Figure 1:**
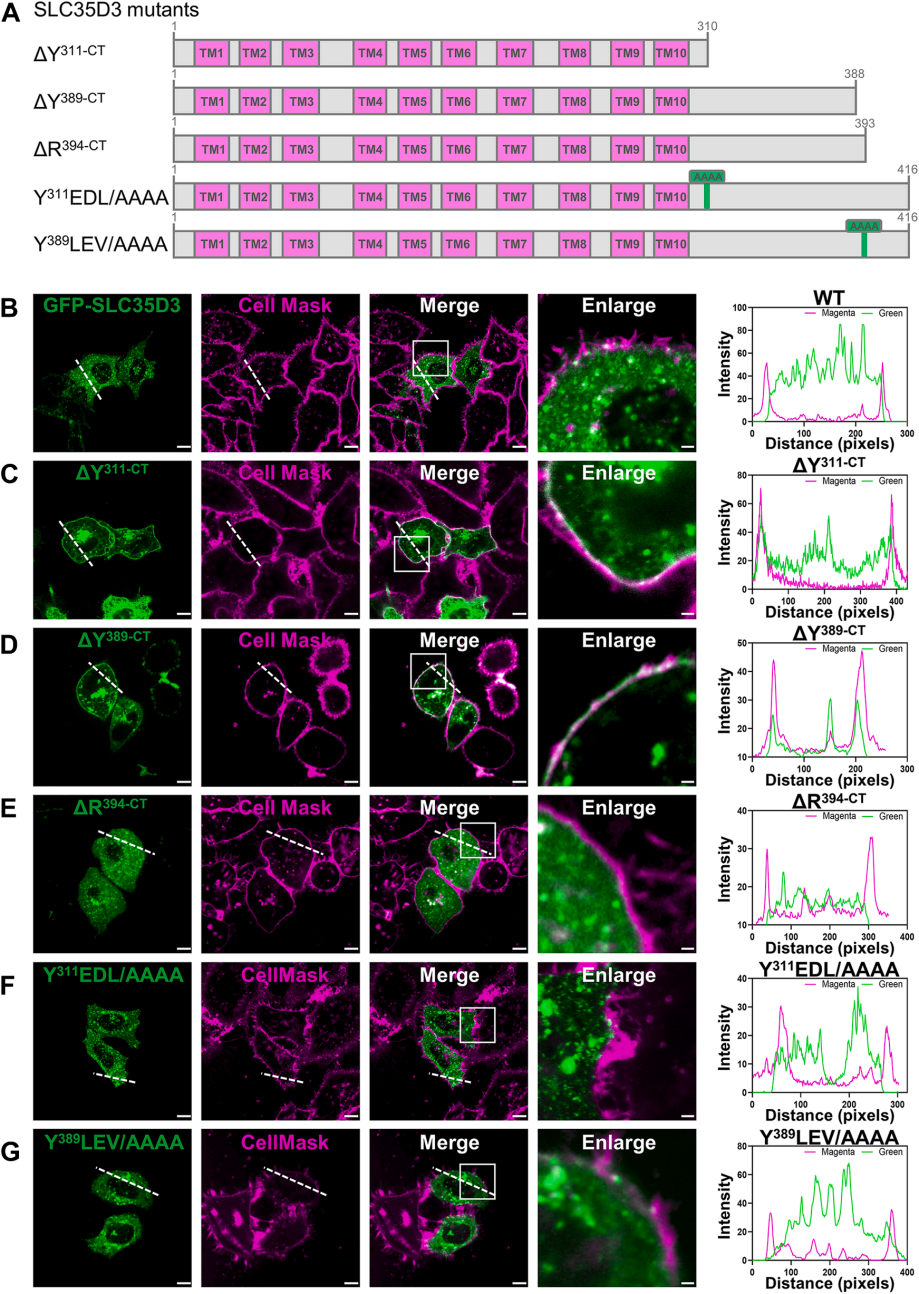
**The C-terminal truncations of SLC35D3, specifically the 1 to 310 (ΔY^311-CT^) and 1 to 388 (ΔY^389-CT^) variants, exhibit PM localization.***A,* the schematic diagram illustrates the C-terminal truncations of SLC35D3, along with the corresponding alanine substitutions within the two tyrosine-based sorting motifs, Y^311^EDL and Y^389^LEV. *B-G,* Live-cell imaging was employed to ascertain the subcellular localization of SLC35D3. HeLa cells were overexpressed with GFP-SLC35D3 (*B*), GFP-SLC35D3-1-310 (*C*, ΔY^311-CT^), GFP-SLC35D3-1-388 (*D*, ΔY^389-CT^), GFP-SLC35D3-1-393 (*E*, ΔR^394-CT^), GFP-SLC35D3 Y^311^EDL/AAAA (*F*), and GFP-SLC35D3 Y^389^LEV/AAAA (*G*) (pseudo color *green*). Subsequently, the cells were stained with Cell Mask (pseudo color *magenta*), a fluorescent dye that binds to the PM. The ΔY^311-CT^ and ΔY^389-CT^ truncations of SLC35D3 aberrantly localized at the PM. Conversely, the ΔR^394-CT^ and the alanine substitution of the potential AP binding motif of Y^311^EDL (Y^311^EDL/AAAA) and Y^389^LEV (Y^389^LEV/AAAA) did not alter SLC35D3 localization to the PM. The white dashed lines demarcate cellular regions within the merged panels, where fluorescence intensities from the *green* and *magenta* channels were quantified. Fluorescence intensities are visualized using the plot profile method in Image J software as shown in the *right panel*. Scale bars represent 10 μm for overview images and 2 μm for magnified sections.

Proteins destined for endosomes and lysosomes are typically routed either directly from the trans-Golgi network (TGN) or internalized from the plasma membrane (PM) *via* endocytosis. Mutations in sorting signals would result in mislocalization of these proteins to the PM (17, 18). To verify the PM internalized signals in SLC35D3, a series of truncations from the C-terminus and alanine substitutions were constructed (Fig. 1*A*). When expressed in HeLa cells, colocalization of SLC35D3 and its mutants with the PM marker Cell Mask was plotted using line-scan profiling plugin of Image J and the percentage of cells presenting PM-localized SLC35D3 and mutations was also calculated (Fig. S1*D*). Compared to the wild-type (WT) GFP-SLC35D3 (Fig. 1*B*), truncations of SLC35D3 at residues 310 (ΔY^311-CT^) and 388 (ΔY^389-CT^) resulted in prominent PM mislocalization (Fig. 1, *C* and *D*). Instead, the truncation at residue 393 (ΔR^394-CT^) did not affect SLC35D3’s subcellular localization, ruling out the possibility of EVWRL^395^V as a sorting motif (Fig. 1*E*). Thus, the potential sorting signals for SLC35D3 appear to be restricted to the two tyrosine-based motifs, Y^311^EDL and Y^389^LEV, situated at the C-terminus.

**Figure 2:**
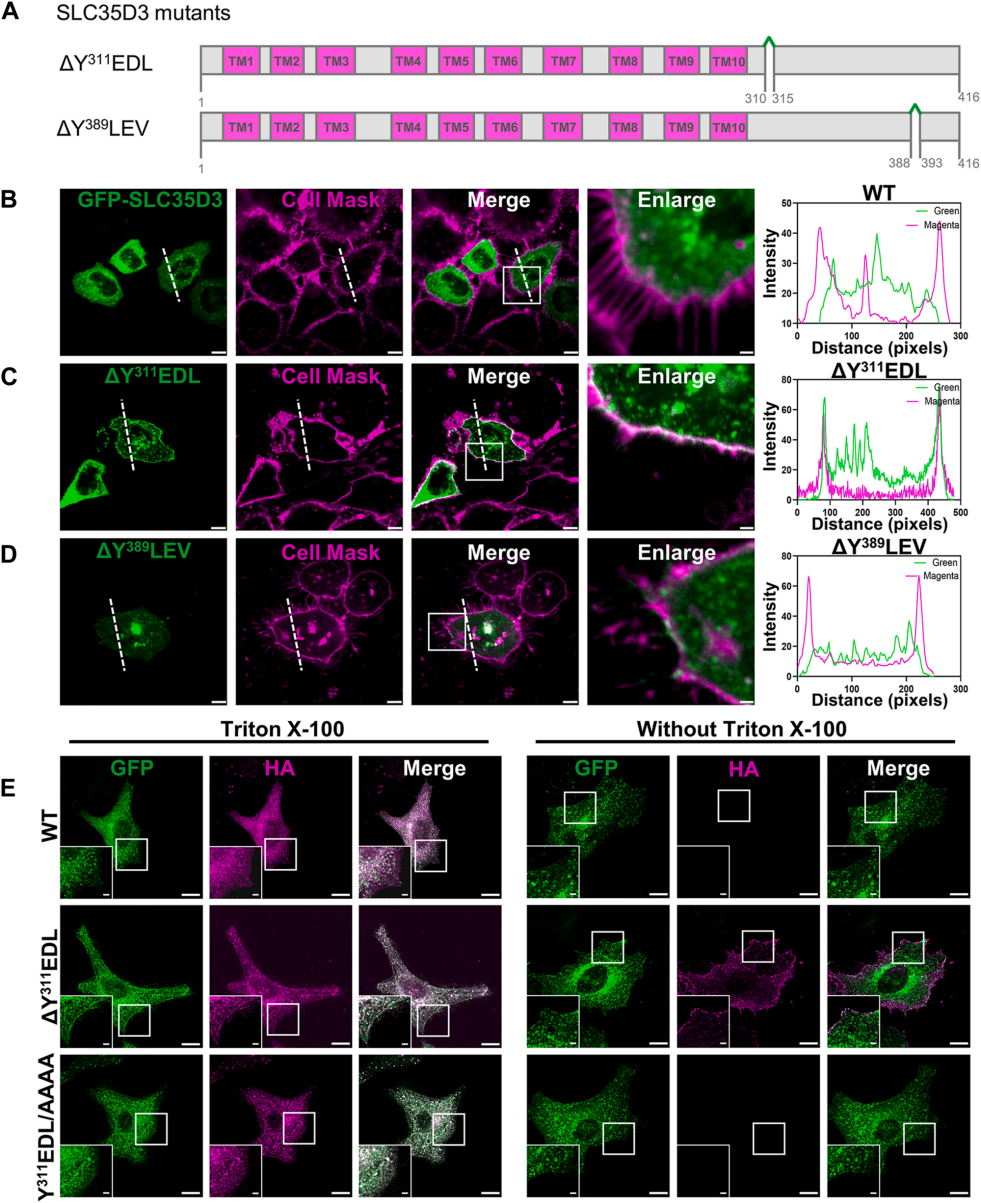
**Aberrant PM localization of SLC35D3 ΔY^311^EDL mutant.***A,* schematic depiction of the secondary structure of full-length and the C-terminus of SLC35D3 ΔY^311^EDL, ΔY^389^LEV deletions. *B-D,* live-cell imaging was employed to ascertain the subcellular localization of SLC35D3. HeLa cells were overexpressed with GFP-SLC35D3 (*B*), GFP-SLC35D3 ΔY^311^EDL (*C*), GFP-SLC35D3 ΔY^389^LEV, and (*D*) (pseudo color *green*). Subsequently, the cells were stained with Cell Mask (pseudo color *magenta*), a fluorescent dye that binds to the PM. Compared with the wide-type SLC35D3, the ΔY^311^EDL deletion mutant of SLC35D3 aberrantly localized at the PM. The white dashed lines delineate the cellular regions within the merged panels where the fluorescence intensities for the *green* and *magenta* channels were quantified. Fluorescence intensity are depicted using the plot profile method in Image J software as shown in the *right panel*. *E,* HeLa cells were overexpressed with WT (GFP-HA-SLC35D3), ΔY^311^EDL (GFP-HA-SLC35D3 ΔY^311^EDL), and Y^311^EDL/AAAA (GFP-HA-SLC35D3 Y^311^EDL/AAAA) mutants. Cells were either fixed directly, followed by permeabilization with 0.05% Triton X-100 (Triton X-100) or fixed without permeabilization (without Triton X-100). All Cells were immunostained with HA antibody (pseudo color *magenta*), and GFP (pseudo color *green*) was visualized. The scale bars correspond to 10 μm for the overview images and 2 μm for the magnified sections.

To further identify the sorting signals, alanine scanning mutagenesis was performed, in which all residues within the putative sorting motifs were substituted with alanines, to study the roles of Y^311^EDL and Y^389^LEV in the internalization of SLC35D3 from the PM. Unexpectedly, we found that the alanine substitution of all the amino acids in either of these two motifs did not cause PM localization (Fig. 1, *F* and *G*). Therefore, we attempted to delete the potential sorting signals (Fig. 2*A*). Compared to the WT GFP-SLC35D3 (Fig. 2*B*; Fig. S1*E*), only the Y^311^EDL deletion (ΔY^311^EDL), rather than ΔY^389^LEV, led to mislocalization to the PM (Fig. 2, *C* and *D*; Fig. S1*E*). To further evaluate the effects of sorting signals on PM localization, we engineered an HA-tag into the ninth loop of SLC35D3 that would face the extracellular side of the cell when it be translocated to the PM (Fig. S2*A*). The detection of HA tag by HA antibody without Triton X-100 treatment would reflect the amount of PM-localized SLC35D3. With this surface labeling method, we found that SLC35D3-ΔY^311^EDL was specifically recognized on the PM, while the Y^311^EDL/AAAA and WT did not (Fig. 2*E*; Fig. S2*B*), which is consistent with the above results (Fig. 1, *D* and *E*). This suggests that ΔY^311^EDL disrupts a sorting signal that is not a canonical tyrosine-based motif.

Notably, the fluorescence of the ΔY^389^LEV mutant was found in the perinuclear region, which suggests that its trafficking out of the TGN may be compromised. We therefore analyzed its colocalization with the Golgi apparatus marker mRuby2-GS28 in HeLa cells. Compared to the WT GFP-SLC35D3, ΔY^389^LEV, ΔY^311-CT^ and ΔY^389-CT^ showed significantly more pronounced localization at the Golgi apparatus (Fig. S2, *C* and *D*). However, it remains unclear whether this retention is mediated by AP-1 regulation of SLC35D3 trafficking.

### The deletion of Y^311^EDL perturbs SLC35D3 binding to AP-3

Tetrameric clathrin adaptor proteins play an important role in sorting proteins from the Golgi to intracellular destinations (10, 19). Therefore, we tested if mutation of Y^311^EDL disrupted SLC35D3 binding to AP-1, AP-2, or AP-3, which are likely involved in SLC35D3 intracellular sorting.

**Figure 3:**
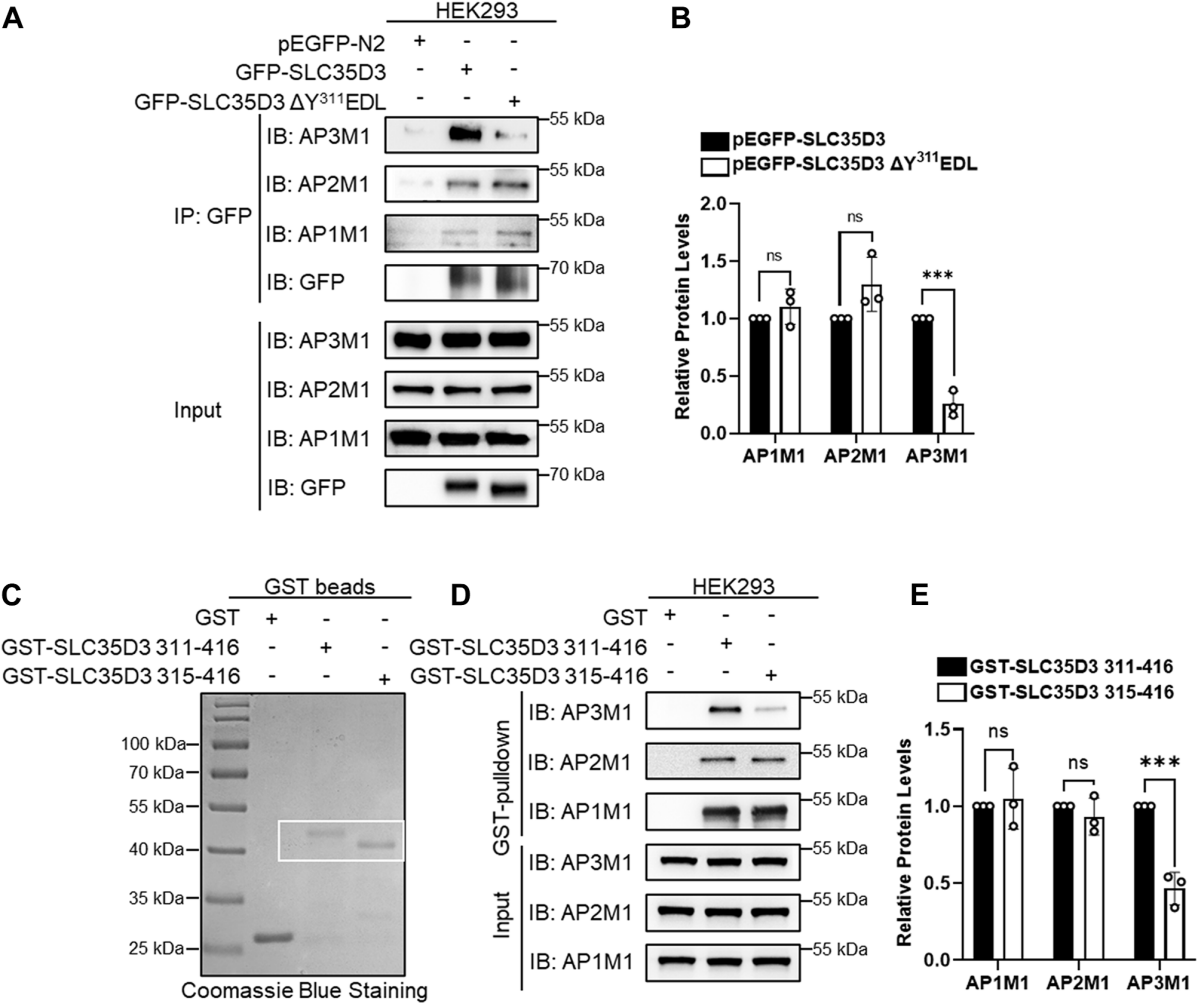
**The deletion of Y^311^EDL perturbs SLC35D3 binding to AP-3.***A,* Co-IP assays were performed to ascertain the association between overexpressed GFP-SLC35D3 and GFP-SLC35D3 ΔY^311^EDL with the endogenous AP1M1 (AP1M1), AP-2 (AP2M1) and AP-3 (AP3M1) complexes. The results indicated that the absence of the Y^311^EDL motif in SLC35D3 did not alter the binding affinity with AP-1 or AP-2. But Y^311^EDL deletion reduced the association between SLC35D3 and AP-3. *B,* Quantification analysis of AP1M1, AP2M1 and AP3M1 band intensities pulled down by the WT *versus* ΔY^311^EDL constructs from three independent biological replicates demonstrated a statistically significant reduction in AP3M1 binding by the ΔY^311^EDL construct compared to WT (0.26 ± 0.11, n = 3, *p* < 0.001), confirming that deletion of the Y^311^EDL motif consistently impaired binding to AP-3 μ subunit. *C,* Coomassie blue staining of purified GST fusion proteins *in vitro*. Protein samples loaded were indicated on top of each lane. White squares marked the band of the bait proteins. *D* and *E,* GST pull-down assays were performed to ascertain the physical interaction between the glutathione S-transferase (GST)-tagged C-terminal cytoplasmic domain of SLC35D3 (encompassing the Y^311^EDL motif and its flanking cytoplasmic sequences) and AP complexes. Quantification analysis of AP1M1, AP2M1 and AP3M1 band intensities demonstrated a statistically significant reduction of bound AP3M1 by the cytosolic tail without the Y^311^EDL motif (315–416) compared to the cytoplasmic tail of SLC35D3 containing the intact Y^311^EDL motif (311–416) (0.46 ± 0.11, n = 3, *p* < 0.001). The absence of the Y^311^EDL motif in SLC35D3 did not alter the binding affinity for the μ subunit of AP-1 and AP-2 complexes. IP assays were performed using antibodies against AP1M1, AP2M1 and AP3M1. The full-length SLC35D3 and the cytoplasmic tail of SLC35D3 containing the intact Y^311^EDL motif values were normalized to a baseline of 1. Data are presented as mean ± SD. Statistical significance was assessed by two-tailed unpaired *t* test, ns, *p* > 0.05, ∗∗∗*p* < 0.001.

Our co-IP assays demonstrated that only the deletion of the Y^311^EDL motif compromised the association between SLC35D3 and AP-3, but the single-point substitutions at Y^311^ (Y^311^/A and Y^311^/F) and the tandem alanine substitution of the motif (Y^311^EDL/AAAA) did not impact AP-3 binding (Fig. S2*, E* and *F*). To explore whether the Y^311^EDL motif was recognized by AP-1, AP-2, or AP-3, GFP-SLC35D3 ΔY^311^EDL was overexpressed in HEK293 cells to evaluate the interaction with endogenous μ subunits of AP-1∼3. Immunoprecipitation (IP) assays revealed that SLC35D3 associated with the endogenous μ1 (AP1M1), μ2 (AP2M1), and μ3 (AP3M1) subunits of the AP-1, AP-2, and AP-3 adaptor complexes, respectively. The SLC35D3 ΔY^311^EDL did not impact the binding affinity for the endogenous μ subunit of AP-1 and AP-2 complexes. In contrast, SLC35D3 ΔY^311^EDL exhibited a significant reduction in its association with the endogenous μ3 subunit of AP-3 (Fig. 3, *A* and *B*).

We further performed GST-pulldown assays to ascertain the interaction between the glutathione S-transferase (GST)-tagged C-terminal cytoplasmic domain of SLC35D3 and AP complexes. The results indicated that the cytoplasmic tail of SLC35D3 containing the intact Y^311^EDL motif robustly pulled down the μ3 subunit of AP-3. In contrast, the cytosolic tail without the Y^311^EDL motif showed markedly reduced binding. However, the absence of the Y^311^EDL motif in the SLC35D3 C-tail did not alter the binding affinity for the μ subunit of AP-1 and AP-2 complexes (Fig. 3, *C*–*E*). This suggests that the integrity of the Y^311^EDL motif is important rather than the identity of any single residue within it. These data support that the Y^311^EDL stretch is required for SLC35D3 binding to AP-3.

**Figure 4:**
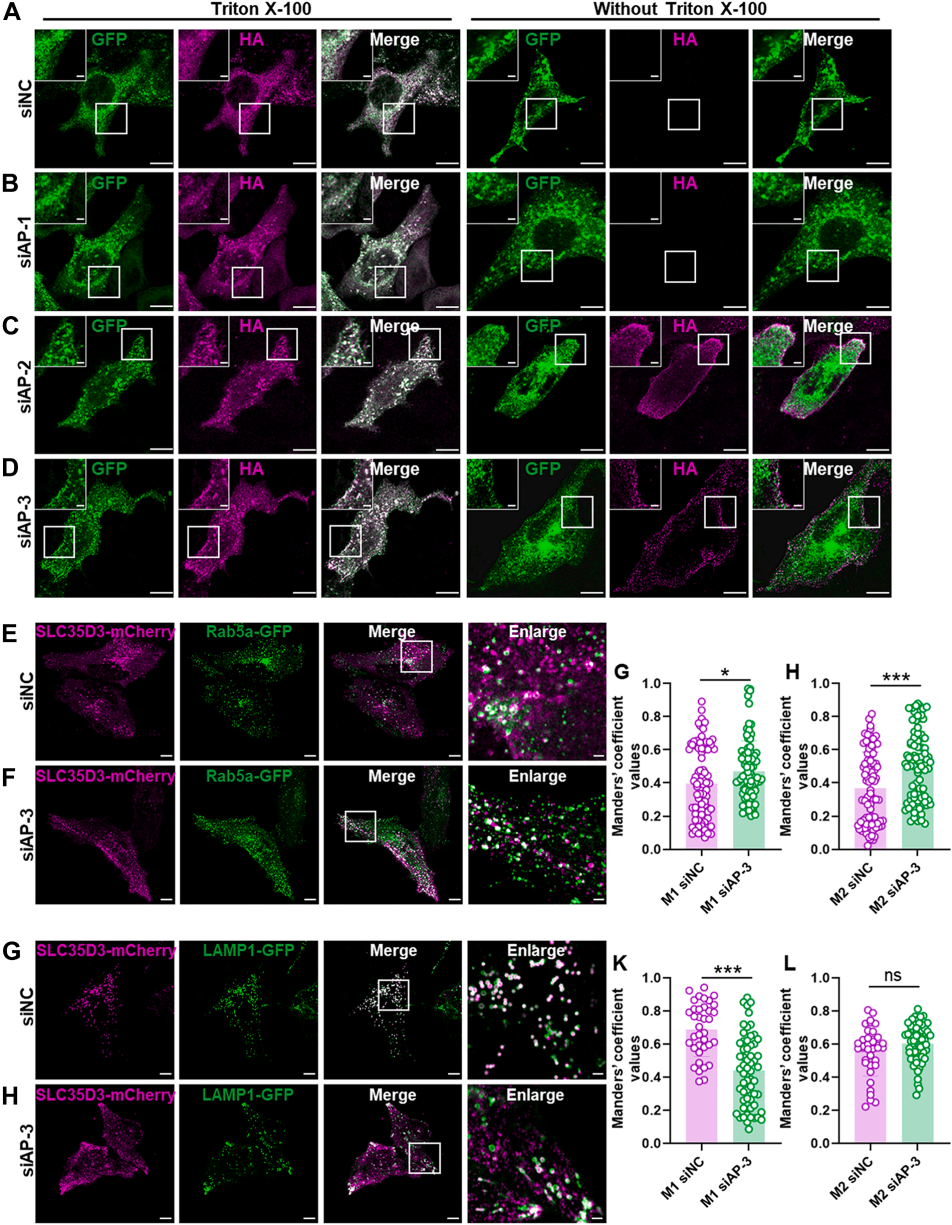
**Altered subcellular localization of SLC35D3 in AP-3 knockdown cells.***A-D,* PM localization of SLC35D3 in AP complexes knockdown cells. HeLa cells were overexpressed with WT (GFP-HA-SLC35D3) and subjected to knockdown of different adaptor protein (AP) complexes. Cells were either fixed directly and then permeabilized with 0.05% Triton X-100 (Triton X-100) or fixed without permeabilization (without Triton X-100). Immunofluorescence staining was performed using antibodies against HA to ascertain the localization of SLC35D3 following knockdown of AP-1 (siAP-1) (*B*), AP-2 (siAP-2) (*C*), or AP-3 (siAP-3) (*D*). SLC35D3 was predominantly localized to the PM (*C* and *D*) compared with the control (siNC) (*A*). Cells were immunostained with HA antibody (pseudo color *red*) and GFP (pseudo color *green*). The scale bars correspond to 10 μm for the overview images and 2 μm for the magnified sections. *E* and *F,* HeLa cells were overexpressed with SLC35D3-mCherry (pseudo color *magenta*) and Rab5a-GFP (early endosome marker) (pseudo color *green*). *G*, Quantification of colocalization coefficient for the distribution of M1 (fraction of SLC35D3 area in Rab5a area). AP-3 knockdown resulted in abnormal accumulation of SLC35D3 in early endosomes (siAP-3, 0.47 ± 0.18, n = 85, *p* < 0.05), compared with non-targeting siRNA control (siNC, 0.40 ± 0.22, n = 82). *H*, Quantification of colocalization coefficient for distribution of M2 (fraction of Rab5a area in SLC35D3 area). AP-3 knockdown resulted in abnormal accumulation of SLC35D3 in early endosomes (siAP-3, 0.50 ± 0.21, n = 85, *p* < 0.001), compared with non-targeting siRNA control (siNC, 0.37 ± 0.22, n = 82). *I* and *J,* HeLa cells were overexpressed with SLC35D3-mCherry (pseudo color *magenta*) and LAMP1-GFP (late endosomes/lysosomes marker) (pseudo color *green*). *K*, Quantification of colocalization coefficient for distribution of M1 (fraction of SLC35D3 area in LAMP1 area). Compared with non-targeting siRNA control (siNC, 0.69 ± 0.16, n = 37), AP-3 knockdown significantly reduced the localization of SLC35D3 to late endosomes/lysosomes (siAP-3, 0.44 ± 0.22, n = 59, *p* < 0.001). *L*, Quantification of colocalization coefficient for distribution of M2 (fraction of LAMP1 area in SLC35D3 area). (siAP-3: 0.60 ± 0.11, n = 59, *versus* siNC: 0.56 ± 0.15, n = 37, *p* = 0.0832). The enlarged pictures showed magnified images of the boxed region. Scale bars represent 10 μm for overview images and 2 μm for magnified sections. Quantification of colocalization using Manders’ colocalization coefficient. The siNC values were normalized to a baseline of 1. Data are presented as mean ± SD. Statistical significance was assessed by two-tailed unpaired *t* test, ns, *p* > 0.05, ∗*p* < 0.05, ∗∗∗*p* < 0.001.

To further elucidate the roles of AP-1, AP-2, and AP-3 in the internalization of GFP-SLC35D3 from the PM, cells were transfected with siRNAs targeting each respective adaptor protein complex (Fig. S3, *F*–*H*). Under these conditions, GFP-SLC35D3 with the engineered HA-tag (Fig. S2*A*) was more retained on the PM of cells with siAP-2 and siAP-3 rather than siAP-1 (Fig. 4, *A*–*D;* Fig. S3, *A*–*E*). Furthermore, HeLa cells were overexpressed with SLC35D3-mCherry and Rab5a-GFP (early endosome marker, EE) or LAMP1-GFP (late endosome/lysosome marker, LE/LYS). Compared with the negative control (siNC), SLC35D3 accumulated in EE (Fig. 4, *E–H*) and reduced LE/LYS localization in siAP-3 cells (Fig. 4, *I*–*L*). These findings suggest that AP-2 mediates the internalization of SLC35D3, whereas AP-3 may be involved in trafficking SLC35D3 from EE to LE/LYS.

### SLC35D3 associates with BLOC-1 in an AP-3 dependent manner

**Figure 5:**
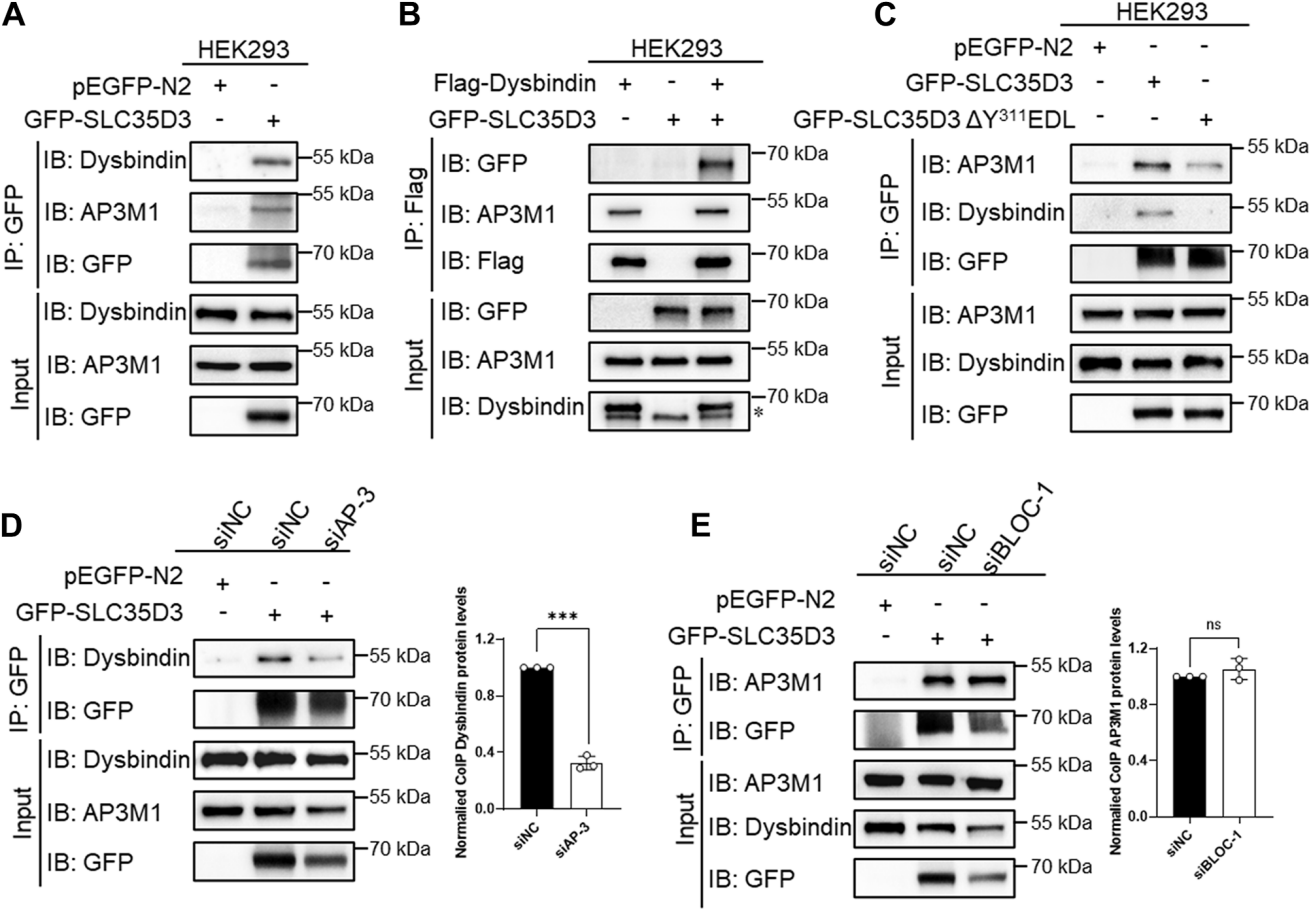
**SLC35D3 associates with BLOC-1 in an AP-3 dependent manner.***A,* IP assays showed overexpressed GFP-SLC35D3 associated with the endogenous Dysbindin subunit of BLOC-1 and μ subunit of AP-3 (AP3M1). *B,* IP assays showed that overexpressed Flag-Dysbindin associated with GFP-SLC35D3. The endogenous AP-3 co-precipitates with Dysbindin, serving as an internal positive control for the integrity of the IP assays. The star sign (∗) represents endogenous Dysbindin. *C,* Co-IP assays showed that the absence of the Y^311^EDL motif (ΔY^311^EDL) in GFP-tagged SLC35D3 weakened the binding affinity for endogenous AP-3 and abolished the association with Dysbindin. *D,* IP assays determined that the association between overexpressed GFP-SLC35D3 and endogenous Dysbindin was weakened after AP-3 complex knockdown (siAP-3, 0.33 ± 0.05, n = 3, *p* < 0.001). Quantitative analysis of the IP results is shown in the *right panel*. *E,* IP assays showed that the interaction between overexpressed GFP-SLC35D3 and endogenous AP-3 was unaltered after Dysbindin knockdown (siBLOC-1, 1.05 ± 0.08, n = 3, *p* = 0.2813). Quantitative analysis of the IP results is shown in the right panel. IP was performed using antibodies against GFP, AP3M1, Dysbindin. The siNC values were normalized to a baseline of 1. Data are presented as mean ± SD. Statistical significance was assessed by two-tailed unpaired *t* test, ns, *p* > 0.05, ∗∗∗*p* < 0.001.

In addition to AP complexes, BLOC-1, BLOC-2, and BLOC-3 were also involved in endolysosomal trafficking, thereby playing crucial roles in the biogenesis of melanosomes and platelet DGs (11). BLOC-1 was shown to engage in both physical and functional interactions with the AP-3 complex, thus facilitating the trafficking of the AP-3 cargo CD63 (20). BLOC-1 deficiency led to a reduction of SLC35D3, implicating a role of BLOC-1 in SLC35D3 trafficking (4). In HEK293 cells, the association between SLC35D3 and subunits from the two distinct protein complexes, specifically Dysbindin of BLOC-1 and AP3M1 of AP-3, was identified (Fig. 5*A*). Dysbindin associated with both SLC35D3 and AP3M1, suggesting these three proteins and their associated protein complexes may form a macro-complex during endosomal trafficking (Fig. 5*B*).

To further ascertain the associations of these three proteins, we found that the ablation of the Y^311^EDL motif of SLC35D3 resulted in attenuated interaction with AP3M1 and abolished interaction with Dysbindin (Fig. 5*C*). Furthermore, we observed a significant reduction of association between SLC35D3 and Dysbindin in AP-3 knockdown cells (siAP-3) (Fig. 5*D*). In contrast, knockdown of BLOC-1 (siBLOC-1) did not affect the association between SLC35D3 and AP3M1 (Fig. 5*E*). These data suggest that SLC35D3 ΔY^311^EDL perturbs associations with both AP3M1 and Dysbindin, and the association between SLC35D3 and Dysbindin may depend on the association between SLC35D3 and AP3M1, which requires the Y^311^EDL stretch, thereby promoting the trafficking of SLC35D3 to endolysosomal compartments.

### Y^311^EDL is involved in the sorting of SLC35D3 into DG precursors

**Figure 6:**
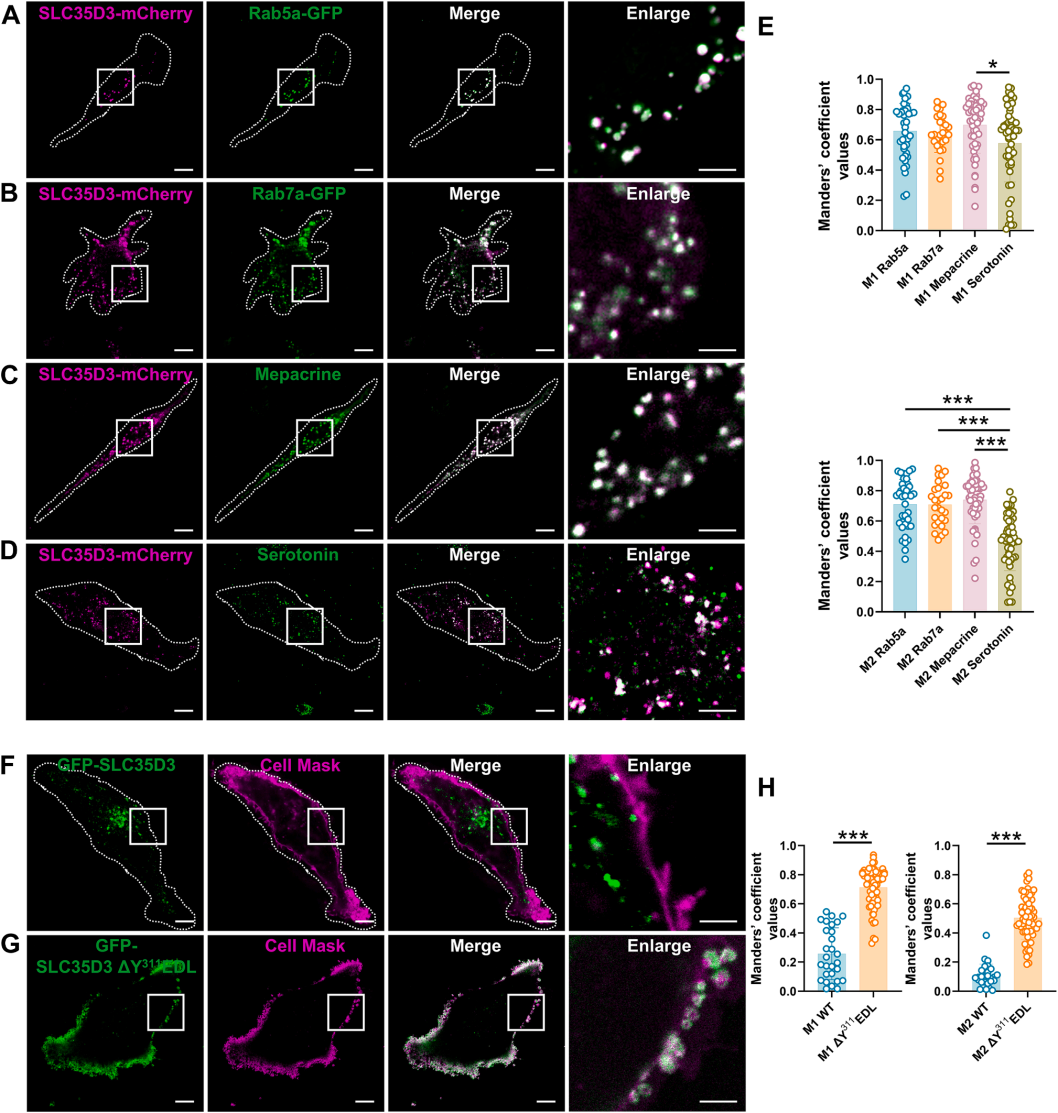
**SLC35D3 localizes to the endosomes and platelet DG precursors, whereas the Y^311^EDL deletion mutant exhibits aberrant localization to the PM in MEG-01 cells.***A-D,* MEG-01 cells were transduced with lentiviral vectors encoding SLC35D3-mCherry. Cells co-expressing SLC35D3-mCherry with Rab5a-GFP (early endosome marker, *A*) or Rab7a-GFP (late endosome marker, *B*) were analyzed by confocal microscopy. For live-cell imaging, cells were stained with mepacrine (*C*) to visualize DG precursors. Immunofluorescence staining was performed on MEG-01 cells expressing SLC35D3-mCherry using antibodies against serotonin to confirm the colocalization (*D*). *E*, Quantification of colocalization coefficients for distribution of M1: fraction of SLC35D3 area in Rab5a area (0.66 ± 0.18, n = 40), fraction of SLC35D3 area in Rab7a area (0.64 ± 0.12, n = 28), and fraction of SLC35D3 area in Mepacrine area (0.70 ± 0.19, n = 54). Notably, the colocalization between SLC35D3 and Mepacrine-positive vesicles was slightly higher than that observed with Serotonin-stained vesicles (0.58 ± 0.26, n = 55, *p* < 0.05). Quantification of colocalization coefficients for distribution of M2: fraction of Rab5a area in SLC35D3 area (0.71 ± 0.16, n = 40, *p* < 0.001), fraction of Rab7a area in SLC35D3 area (0.70 ± 0.14, n = 28, *p* < 0.001), and fraction of Mepacrine area in SLC35D3 area (0.74 ± 0.16, n = 54, *p* < 0.001) exhibited significantly higher colocalization coefficients than Serotonin overlapping SLC35D3 (fraction of Serotonin area in SLC35D3 area; 0.46 ± 0.19, n = 55). Data are presented as mean ± SD. Statistical significance was assessed by one-way analysis of variance (ANOVA) for multiple group comparisons, ns, *p* > 0.05, ∗*p* < 0.05, ∗∗∗*p* < 0.001. *F* and *G,* MEG-01 cells were transfected with lentivirus vectors encoding GFP-SLC35D3 (*F*) and GFP-SLC35D3 ΔY^311^EDL (*G*). *H*, Manders’ colocalization coefficients revealed significant overlapping difference between wild-type (WT) and ΔY^311^EDL mutant SLC35D3 with the plasma membrane (PM): M1 (fraction of SLC35D3 area in PM area) was 0.26 ± 0.18 (n = 28) in WT *versus* 0.71 ± 0.13 (n = 69) in ΔY^311^EDL (*p* < 0.001); M2 (fraction of PM area in SLC35D3 area) was 0.11 ± 0.08 (n = 28) in WT *versus* 0.50 ± 0.15 (n = 69) in ΔY^311^EDL (*p* < 0.001). Data are presented as mean ± SD. Statistical significance was assessed by two-tailed unpaired *t* test, ∗∗∗*p* < 0.001. For live cell imaging, the cells were subsequently stained with Cell Mask to delineate the cell membrane. Cell outlines are indicated by white dashed lines. Insets show magnified views of the boxed regions. The scale bars correspond to 5 μm for the overview images and 2 μm for the magnified sections.

To investigate the role of the Y^311^EDL stretch of SLC35D3 in DG targeting, we utilized a human megakaryocytic cell line, MEG-01, which produces DG precursors upon differentiation, to determine the localization of SLC35D3. Lentivirus carrying fluorescent protein-tagged WT and ΔY^311^EDL SLC35D3 constructs were generated and transduced into MEG-01 cells. Our results demonstrated that full-length SLC35D3 exhibited colocalization with markers of EE (Rab5a-GFP, Fig. 6*A*), LE (Rab7a-GFP, Fig. 6*B*), DG precursor (mepacrine in Fig. 6*C* and serotonin in Fig. 6*D*). Manders’ colocalization analysis of SLC35D3 with these organelle markers has been performed (Fig. 6*E*). The subcellular distribution of WT SLC35D3 was found to be distinct from that of the PM marker Cell Mask (Fig. 6*F*). However, SLC35D3 ΔY^311^EDL led to a marked translocation of SLC35D3 to the PM, indicating that this motif is critical for the intracellular targeting of SLC35D3 to DG precursors (Fig. 6*G*). Manders’ colocalization analysis of SLC35D3 with the PM has been performed (Fig. 6*H*). Taken together, our findings demonstrated that the Y^311^EDL stretch of SLC35D3 is required for SLC35D3 binding to AP-3 and thereafter associated with the BLOC-1 complex for subsequent trafficking to DG precursors, where it functions in DG biogenesis.

### Mislocalized SLC35D3 undergoes lysosomal degradation

**Figure 7:**
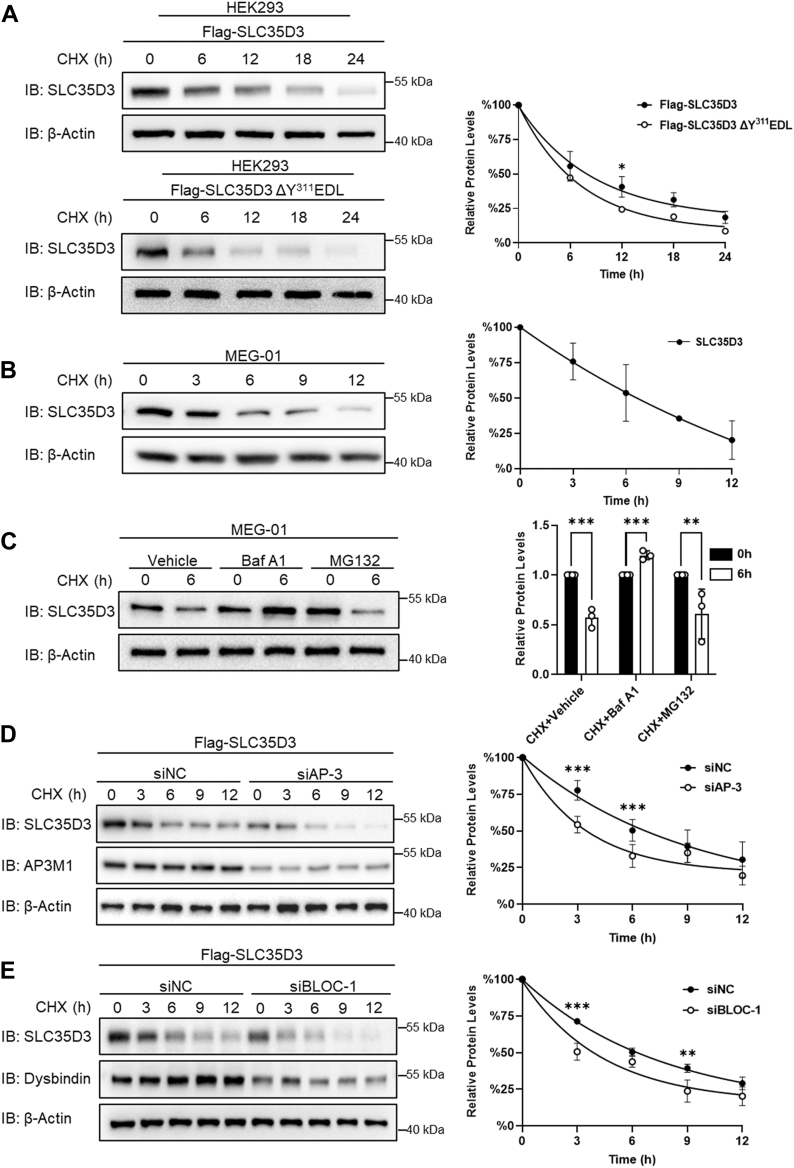
**SLC35D3 is degraded *via* the lysosomal pathway, and the absence of AP-3 or BLOC-1 leads to decreased stability of the SLC35D3 protein.***A,* HEK293 cells expressing the WT SLC35D3 (Flag-SLC35D3) or the ΔY^311^EDL mutant (Flag-SLC35D3 ΔY^311^EDL) were treated with 100 μg/ml cycloheximide (CHX) for indicated time periods. SLC35D3 protein levels were examined by immunoblotting using an anti-SLC35D3 antibody, with β-actin as a loading control. Relative SLC35D3 protein levels were quantified and normalized to β-actin. The half-life of Flag-SLC35D3 was calculated to be approximately 6.00 h, while that of Flag-SLC35D3 ΔY^311^EDL was 4.92 h. Statistical difference was observed at 12 h (WT: 40.73% ± 5.23% *versus* ΔY^311^EDL: 24.39% ± 0.01%, *p* < 0.05). There were no significant difference at 6 h (WT: 55.69% ± 8.80% *versus* ΔY^311^EDL: 47.40% ± 0.89%, *p* = 0.2212); 18 h (WT: 31.38% ± 3.63% *versus* ΔY^311^EDL: 19.06% ± 0.41%, *p* = 0.0592) and 24 h (WT: 18.52% ± 3.11% *versus* ΔY^311^EDL: 8.66% ± 0.18%, *p* = 0.2450). Deletion of the YEDL motif accelerated the turnover of SLC35D3 protein, suggesting that mislocalization of the ΔY^311^EDL mutant contributes to enhanced degradation of SLC35D3. *B,* The half-life of endogenous SLC35D3 was determined following treatment with 100 μg/ml CHX. The half-life time of endogenous SLC35D3 in MEG-01 cells was approximately 5.32 h. *C,* in MEG-01 cells, endogenous SLC35D3 was subjected to treatment with 100 μg/ml CHX in the presence of either the lysosomal inhibitor 100 nm Bafilomycin A1 (BafA1) or the proteasome inhibitor MG132 (20 μm). SLC35D3 protein levels were examined by immunoblotting using an anti-SLC35D3 antibody, with β-actin as a loading control. Quantitative analyses of SLC35D3 protein levels were performed. Treatment of MEG-01 cells with CHX for 6 h resulted in an approximately 50% reduction in endogenous SLC35D3 protein levels. Notably, BafA1 treatment resulted in substantial accumulation of SLC35D3 (19.7% increase at 6 h *versus* 0 h, n = 3), whereas the proteasome inhibitor MG132 failed to restore SLC35D3 from CHX-mediated degradation (39.2% decrease at 6 h *versus* 0 h, n = 3). Collectively, these data indicated that SLC35D3 was degraded *via* a lysosome-dependent pathway. *D* and *E,* the CHX chase assays were performed in HEK293 cells co-transfected with Flag-SLC35D3 and siRNA targeting AP-3 (siAP-3) or BLOC-1 (siBLOC-1). The knockdown of AP-3 and BLOC-1 compromised the degradation pathway of SLC35D3. The ectopically expressed SLC35D3 protein level was significantly reduced in AP-3 knockdown cells, with an approximate half-life of 2.41 h (*D*). For AP-3 knockdown, significantly reduced SLC35D3 levels were observed at 3 h (siNC: 77.69% ± 6.00% *versus* siAP-3: 54.33% ± 4.95%, *p* < 0.001); 6 h (siNC: 50.40% ± 6.76% *versus* siAP-3: 32.99% ± 7.28%, *p* < 0.001). There were no significant difference at other time point (9 h: siNC: 39.58% ± 10.42% *versus* siAP-3: 35.08% ± 5.96%, *p* = 0.8296; 12h: siNC: 30.43% ± 11.18% *versus* siAP-3: 19.57% ± 5.79%, *p* = 0.0585). In cells subjected to BLOC-1 knockdown, the ectopically expressed SLC35D3 protein exhibited a significant decrease at 3 h (siNC: 71.39% ± 1.72% *versus* siBLOC-1: 50.65% ± 6.29%, *p* < 0.001) and 9 h (siNC: 39.40% ± 4.33% *versus* siBLOC-1: 23.77% ± 6.16%, *p* < 0.01), with a half-life of approximately 3.02 h (*E*). There were no significant difference at 6 h (siNC: 50.42% ± 9.61% *versus* siBLOC-1: 43.80% ± 6.20%, *p* = 0.2831) and 12 h (siNC: 29.01% ± 8.32% *versus* siBLOC-1: 20.26% ± 4.58%, *p* = 0.1497). Data are presented as mean ± SD, n = 3. Statistical significance was assessed by unpaired *t* test and two-way analysis of variance (ANOVA) for multiple group comparisons, ∗*p* < 0.05, ∗∗*p* < 0.01, ∗∗∗*p* < 0.001.

It has been shown that the steady-state level of SLC35D3 protein was reduced in platelets of AP-3-deficient *pearl* (*pe*) mice as well as BLOC-1-deficient *pallid* (*pa*) mice, underscoring the critical role of AP-3 and BLOC-1 in regulating SLC35D3 protein levels (4). This observation prompted us to explore how SLC35D3 was destabilized when AP-3 or BLOC-1 was deficient. We assessed the stability of the full-length WT and ΔY^311^EDL epitope-tagged SLC35D3 protein by treating cells with cycloheximide (CHX) to inhibit protein synthesis. WT SLC35D3 exhibited relative stability, with a calculated half-life of approximately 6.00 h, whereas the half-life of ΔY^311^EDL was determined to be 4.92 h (Fig. 7*A*), implicating that the ΔY^311^EDL mutant exhibited slightly accelerated protein degradation kinetics compared to the WT SLC35D3. Similarly, the half-life of endogenous SLC35D3 in MEG-01 cells was approximately 5.32 h (Fig. 7*B*).

**Figure 8:**
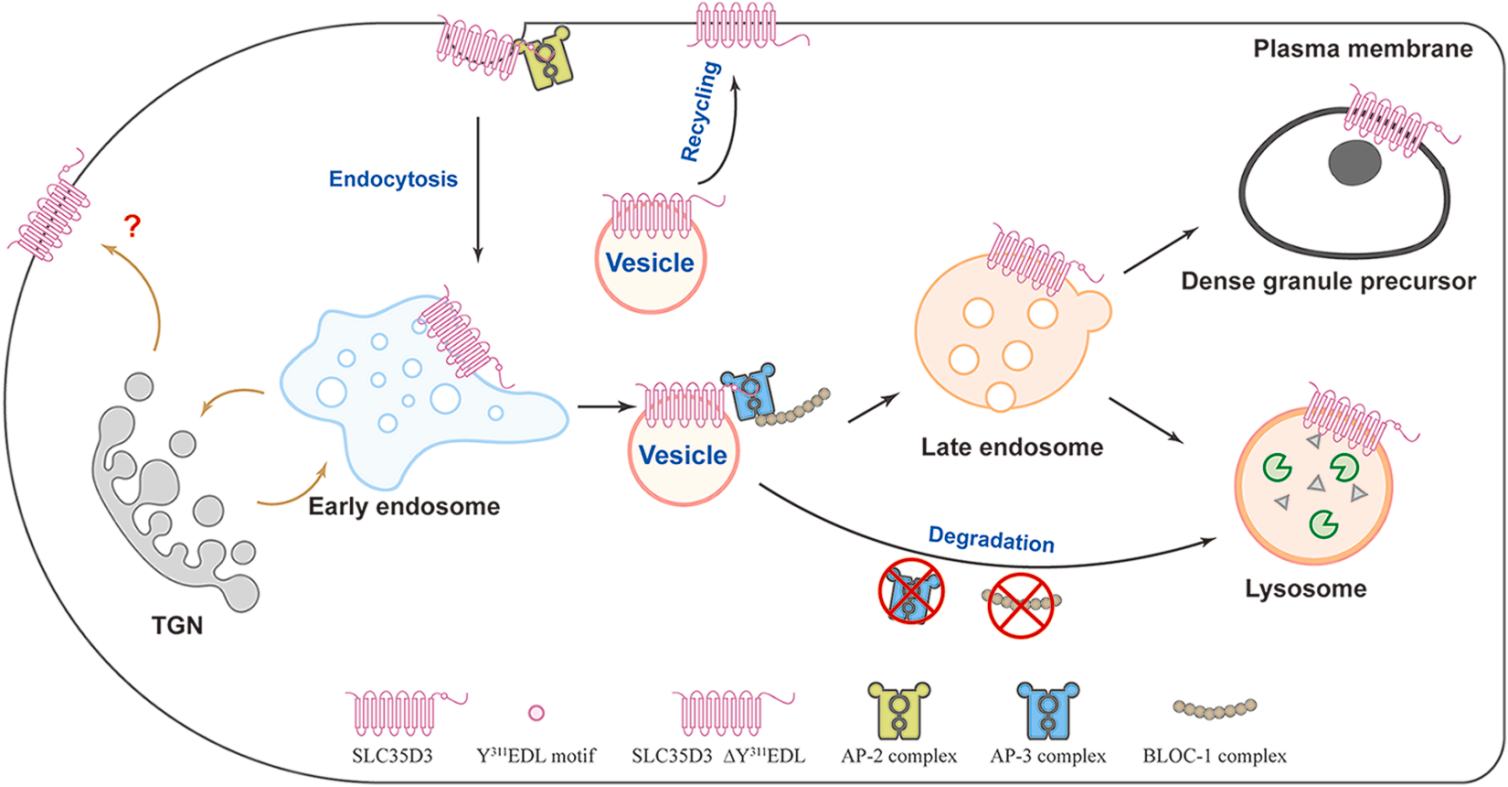
**A model of SLC35D3 trafficking into the DG precursors in megakaryocytes.** Two intracellular pathways may exist in membrane protein sorting into the endolysosomal compartments. The “direct” route transports cargo from the TGN to the endolysosomal compartments by AP-1 or AP-3 buds. The “indirect” route initiates at the TGN and enroutes through the PM *via* AP-2 mediated endocytosis and subsequently transports to endolysosomes/LROs *via* AP-3 using BLOC-1 dependent or independent pathway. Here we show that the Y^311^EDL stretch is required for SLC35D3 binding to AP-3 for its sorting into the DG precursors dependent on BLOC-1. During SLC35D3 trafficking, deficiency in AP-2 may lead to PM accumulation *via* the “indirect” route; deficiency in AP-3 may lead to the accumulation in the early endosome *via* the “direct” route, which may enroute to the PM *via* the recycling endosome. Deficiency in AP-3 or BLOC-1 leads to lysosomal degradation of SLC35D3, and lack of SLC35D3 in DGs causes defective DG biogenesis and function.

To further investigate the pathway involved in SLC35D3 degradation, we measured the rate of SLC35D3 degradation in MEG-01 cells in the presence of various protease inhibitors. When cells were treated with CHX in the presence of the lysosomal inhibitor bafilomycin A1 (BafA1), substantial accumulation of SLC35D3 was observed. In contrast, only a modest amount of SLC35D3 accumulated when the proteasome-specific inhibitor MG132 was applied (Fig. 7*C*). These data suggest that the degradation of SLC35D3 was mainly through the lysosomal degradation pathway. In cells with AP-3 or BLOC-1 knockdown, ectopically expressed SLC35D3 was dramatically decreased, with half-lives of approximately 2.41 h and 3.02 h, respectively (Fig. 7, *D* and *E*). The knockdown efficiency for AP-3 and BLOC-1 was assessed (Fig. S3, *I* and *J*). These findings suggested that the mistargeted SLC35D3 protein undergoes lysosome-dependent degradation when AP-3 or BLOC-1 is deficient (A model in Fig. 8).

## Discussion

This study has determined that deletion of Y^311^EDL disrupts an undefined sorting signal within SLC35D3 that is pivotal for intracellular trafficking and association with the AP-3 complex. SLC35D3 undergoes sequential endolysosomal trafficking mediated by AP-3 and BLOC-1, ultimately targeting platelet DG precursors. The mis-targeted protein could be degraded through the lysosomal pathway. Our findings have elucidated the mechanism underlying the endolysosomal trafficking of SLC35D3 to DGs.

It has been demonstrated that SLC35D3 regulates the function of platelet DGs (3). When ectopically expressed, SLC35D3 is predominantly localized to endolysosomal compartments and can also be directed to LROs. This trafficking process requires the involvement of the HPS protein associated complex AP-3 and BLOC-1 (4). The SLC35D3 sorting mechanism is different from several other LRO cargo sorting di-leucine (LL) motifs recognized by AP-1 or AP-3. The ATP8A1 trafficking to lamellar bodies (LBs) is mediated by an AP-3 recognized LL motif. The disruption of this binding causes ATP8A1 accumulation in early or recycling endosomes (21). Tyrosinase is sorted into AP-1 or AP-3 endosomal buds and then transported to melanosomes independent of BLOC-1. In contrast, TYRP1 is sorted into AP-1 buds (but not AP-3) and then to mature melanosomes mediated by BLOC-1 (6, 18). Similar to SLC35D3, OCA2 and TMEM163 are sorted into AP-3 buds and thereafter is directed by BLOC-1 into LROs (15, 22). However, unlike another DG cargo TMEM163, AP-3 and BLOC-1 competitively binds to the L^69^L^70^ motif of TMEM163 in a mutually repulsive manner during sequential endosomal trafficking (15). The association of BLOC-1 with SLC35D3 is dependent on the association between AP-3 and SLC35D3 by likely forming a super-complex for trafficking.

Although tyrosine-based sorting signals canonically rely on the hydroxyl group of the tyrosine residue for direct recognition by the μ subunit of AP complexes, our mutational analyses reveal an unexpectedly complex relationship between sequence composition and functional impact at this motif. Single-residue substitution at Y^311^ (Y^311^/A and Y^311^/F) and complete alanine substitution of the entire motif (Y^311^EDL/AAAA) each failed to impair AP3M1 binding in co-IP assays, whereas deletion of all four residues (ΔY^311^EDL) markedly reduced this association. Although the Y^311^EDL/AAAA variant retained AP-3 binding activity, it did not accumulate in the PM (Figs. 1*F* and 2*E*), suggesting that the amino acid substitution within this stretch may be insufficient to cause abnormal localization to the PM. It seems that this four-residue segment of SLC35D3 is not merely a linear recognition epitope but may serve as a topological domain that positions adjacent determinants for engaging AP-3, while complete deletion of these tandem residues disrupts the local topology of the cytoplasmic tail and abolishes the interaction. Similar to OCA2, in which substitution of both dileucine motifs (LL1 and LL2), rather than one, is required to redirect OCA2 to the PM (23). This observation reflects either the redundancy among multiple sorting signals or the integrity of any sorting motifs is required for proper intracellular sorting. Whether an analogous structural mechanism operates in SLC35D3 remains to be determined.

A plausible model is proposed to define two distinct pathways for the transport of newly synthesized membrane proteins to lysosomes or LROs (Fig. 8). The first pathway is termed the “direct” route, which transports cargo from the trans-Golgi network (TGN) to endosomal compartments by AP-1 or AP-3 and ultimately to lysosomes or LROs. The second pathway is referred to as the “indirect” route, which initiates at the TGN, enroutes through the PM with an unknown mechanism, and subsequently transports to endosomes likely by AP-2 and then lysosomes/LROs *via* AP-3 utilizing BLOC-1 dependent or independent pathway (24). This “indirect” route is evident that mutation of the recognized motif leads to accumulation to the PM. Alternatively, the accumulated cargo on the EE in the “direct” route may en route to the PM *via* the recycling endosome (15). Our findings suggest that deficiency of either AP-3 or BLOC-1 leads to lysosomal degradation of SLC35D3, thus compromising its function in DG biogenesis and homeostasis. The elucidation of SLC35D3 trafficking into the DGs is helpful for the understanding of the pathogenesis of DG defects in several subtypes of HPS.

## Experimental procedures

### Antibodies and reagents

The experiments were conducted using the following antibodies and reagents. A rabbit polyclonal antibody specific for SLC35D3 was from ThermoFisher Scientific. The mouse monoclonal β-Actin antibody, Flag antibody, and M2 affinity gel were from Sigma-Aldrich. The AP1M1 antibody was obtained from Proteintech, while the AP2M1 and AP3M1 antibodies were from Abcam. The GFP was from Easybio. The Dysbindin and HA antibodies were obtained from Cell Signaling Technology. For immunoprecipitation of GFP-fusion proteins, the GFP agarose beads (gta-20) were from Chromotek. GST magnetic beads (20562ES25) were from Yeasen. The cycloheximide (CHX, HY-12320) and MG-132 (HY-13259) were obtained from MedChemExpress. Bafilomycin A1 (88899-55-2) and mepacrine (Q2876) were from Sigma-Aldrich. CellMask Deep Red (C10046) was from ThermoFisher Scientific. Detailed information on antibodies and reagents is shown in Tables S1 and S2.

### Cell culture and transfection

HeLa cells (Meisen, Jinhua, China, CTCC-001-0006) and HEK293 cells (Meisen, CTCC-003-0015) were cultured in Dulbecco’s Modified Eagle Medium (DMEM, Gibco, 11965092). MEG-01 cells (Meisen, CTCC-ZHYC-0299) were maintained in the 1640 medium (RPMI-1640, Sigma-Aldrich, R0883-500ML). Both types of cells were supplemented with 10% fetal bovine serum (FBS, Gibco, 10099141C) and 1% penicillin/streptomycin (Gibco, 15140122). Cells were incubated at 37 °C in a humidified atmosphere containing 5% CO_2_. Transfections were performed using Lipofectamine 3000 (Invitrogen, L3000015) or Polyethylenimine Linear (Yeasen, 40816ES03) reagents when cells reached 70 to 80% confluence. Following incubation for the designated period, the cells were harvested for subsequent experimental analyses.

### DNA constructs, cloning, and site-directed mutagenesis

The plasmids encoding N-terminal GFP-SLC35D3, Flag-SLC35D3, Flag-Dysbindin, C-terminal Rab5a-GFP, Rab7a-GFP, and LAMP1-GFP were preserved in our laboratory collection. The C-terminal SLC35D3-mCherry was derived from the TPC1-mCherry plasmid, a gift from Antony Galione (Addgene plasmid #135182; http://n2t.net/addgene: 135182; RRID: Addgene_135182), with the TPC1 coding sequence replaced by the SLC35D3 using infusion cloning. The pWPLXd-GFP-SLC35D3, pWPLXd-GFP-SLC35D3 ΔY^311^EDL and pWPLXd-SLC35D3-mCherry plasmids were generated from the pWPLXd-TMCO1 plasmid (a gift from Prof. Tieshan Tang’s lab, Institute of Zoology, CAS), with the TMCO1 coding sequence replaced by the GFP-SLC35D3, GFP-SLC35D3 ΔY^311^EDL or SLC35D3-mCherry through the application of infusion cloning. Lentivirus packaging plasmids psPAX2 and pMD2.G were obtained as gifts from Prof. Tieshan Tang’s lab. The N-terminal mRuby2-tagged full-length GS28 (mRuby2-GS28) was obtained as a gift from Prof. Liang Ge’s lab (Tsinghua University).

The coding sequences of AP1M1, AP2M1, and AP3M1 were amplified from HeLa cell cDNA and subsequently cloned into the pCMV-Tag2B expression vector, which incorporates a Flag epitope tag as Flag-AP1M1, Flag-AP2M1, and Flag-AP3M1. SLC35D3 mutants, including GFP-SLC35D3 ΔY^311^EDL, GFP-SLC35D3 ΔY^389^LEV, GFP-SLC35D3 ΔY^311-CT^, GFP-SLC35D3 ΔY^389-CT^, GFP-SLC35D3 ΔR^394-CT^, GFP-SLC35D3 Y^311^EDL/AAAA, GFP-SLC35D3 Y^389^LEV/AAAA, GFP-SLC35D3 Y^311^/A, and GFP-SLC35D3 Y^311^/F were generated by site-directed mutagenesis with a PCR-based approach utilizing the high-fidelity enzyme KOD-Plus-Neo kit (TOYOBO, KOD-401, Japan). The HA tag was cloned into the GFP-SLC35D3, GFP-SLC35D3 ΔY^311^EDL, GFP-SLC35D3 Y^311^EDL/AAAA plasmids, generating GFP-HA-SLC35D3, GFP-HA-SLC35D3 ΔY^311^EDL, and GFP-HA-SLC35D3 Y^311^EDL/AAAA. GST-SLC35D3 with C-terminal fragment including residues 311 to 416 and 315 to 416 were cloned into the pGEX-4T-1 expression vector, which incorporates a GST epitope tag as GST-SLC35D3^311-416^ and GST-SLC35D3^315-416^. All constructs were verified by Sanger sequencing.

### siRNA treatment

The sense strands of the following small interfering RNAs (siRNAs) were synthesized by GenePharma. A total of 20 nM of siRNAs were introduced into HeLa or HEK293 cells utilizing Lipofectamine RNAiMAX transfection reagent (Thermo Fisher Scientific, 13778150), following the manufacturer’s protocol. After transfection, the cells were incubated for 48 h for further experimental procedures. Detailed information of constructs is shown in Table S3.

### Co-immunoprecipitation (Co-IP) assays

HEK293 cells were cultured on 10 cm dishes for 24 h post-transfection and washed sequentially three times with ice-cold phosphate-buffered saline (PBS, Hyclone Cytiva, SH30256.01). The cells were then detached using a cell scraper and lysed in IP lysis buffer (150 mM NaCl, 20 mM Tris, 1 mM EDTA, 1% Triton X-100, pH adjusted to 7.4) supplemented with a protease inhibitor cocktail (Sigma-Aldrich, P8340) on ice for 10 min. Lysates were centrifuged at 4 °C for 30 min at 13,000*g*. A portion of the supernatant was retained as total lysate. The remaining lysate was incubated with either anti-FLAG M2-Agarose affinity gel (Sigma-Aldrich, A2220) or anti-GFP Agarose affinity beads (Chromotek, gta-20) at 4 °C overnight to allow binding of target proteins to the respective affinity beads. The agarose beads were washed three times consecutively with IP lysis buffer, thereby preparing the sample for subsequent immunoblotting analysis. Detailed information of siRNA primer sequences is shown in Table S4.

### GST-pulldown assays

The recombinant plasmid encoding GST-tagged bait protein was transformed into *E. coli* strain and incubated at 37 °C until the OD600 reached 0.6 to 0.8. Protein expression was induced by adding isopropyl β-D-1-thiogalactopyranoside (IPTG) to a final concentration of 0.2 mM, followed by incubation at 16 °C overnight (16–18 h) with gentle shaking. The cultured cells were centrifuged at 6000*g* for 10 min at 4 °C. The pellet was resuspended in 2 ml of ice-cold PBS containing 1% Triton X-100 and 1 mM PMSF. Cells were lysed by sonication on ice (3-s pulses, 5-s intervals, total 10 min) until the lysate became clear. The lysate was centrifuged at 12,000*g* for 30 min at 4 °C. The supernatant was collected and incubated with 50 μl of pre-equilibrated GST magnetic beads at 4 °C overnight with gentle rotation. The beads were washed three times with 1 ml of ice-cold PBS containing 1% Triton X-100. GST magnetic beads (loaded with GST fusion protein or GST alone as a negative control) were incubated with HEK293 cell lysate in 1 ml of IP lysis buffer at 4 °C overnight with gentle rotation. The beads were washed five times with 1 ml of ice-cold PBS containing 1% Triton X-100. After the final wash, all residual buffer was carefully removed without disturbing the beads. Bound proteins were eluted by adding 50 μl of 1 × SDS-PAGE loading buffer and boiling at 95 °C for 10 min. The eluted proteins were separated by SDS-PAGE and analyzed by Coomassie Blue staining or Western blotting.

### Immunoblotting

Protein samples were diluted with protein loading buffer (Beyotime, Beijing, China, P0015L) and then subjected to electrophoresis on 8% or 10% sodium dodecyl sulfate-polyacrylamide gel electrophoresis (SDS-PAGE) gels at a voltage of 90 V. Proteins were transferred onto polyvinylidene difluoride (PVDF) membranes (Millipore, New Bedford, IPVH00010) at a current of 300 mA for 1 h. The PVDF membranes were blocked with PBS containing 0.1% Tween-20 (PBST) and 5% skim milk (BD Biosciences, 232100) for 1 h at room temperature. Following this blocking step, the membranes were washed three times in PBST. Subsequently, they were incubated with specific primary antibodies at 4 °C overnight. After three further washes in PBST, the membranes were incubated with appropriate horseradish peroxidase-conjugated secondary antibodies. The detection of immunoreactive bands was executed using highly sensitive enhanced chemiluminescence (ECL) luminescence reagent (Meilunbio, MA0187).

### Protein secondary structure and evolutionary conservation analysis

Amino acid sequences of SLC35D3 homologs were aligned using the multiple sequence alignment function in Clustal Omega (25). The secondary structure of SLC35D3, including transmembrane (TM) helices (indicated in pink boxes), was analyzed using DeepTMHMM, a deep learning protein language model-based algorithm capable of detecting and predicting the topology of both alpha helical and beta barrel transmembrane domains. Detailed information on web servers is shown in Table S5.

### Protein stability assay

The stability of SLC35D3 in HEK293 and MEG-01 cells was evaluated using a cycloheximide (CHX)-based protein-chase experiment. For HEK293 cells, a plasmid encoding Flag-tagged SLC35D3 (Flag-SLC35D3) or ΔY^311^EDL mutant (Flag-SLC35D3 ΔY^311^EDL) was transfected using Lipofectamine 3000 reagents (Invitrogen, L3000015). Following transfection, the culture medium was replaced with DMEM supplemented with 10% fetal bovine serum and CHX at a concentration of 100 μg/ml. Cells were harvested at various time points post-CHX treatment (0, 6, 12, 18, and 24 h). To assess the stability of endogenous SLC35D3 in MEG-01 cells, cells differentiated for 4 days with 12-O-Tetradecanoylphorbol-13-Acetate (TPA, 4174, Cell Signaling Technology) were subjected to CHX treatment and collected at distinct time intervals (0, 3, 6, 9, and 12 h). The siRNA treatment was administered before the overexpression of Flag-SLC35D3. The Western blot bands were measured using the Image J software.

### Lentivirus packaging

MEG-01 cells were transiently transduced with either pWPLXd-GFP-SLC35D3, pWPLXd-GFP-SLC35D3 ΔY^311^EDL or pWPLXd-SLC35D3-mCherry plasmids, along with a lentivirus packaging mix containing psPAX2 and pMD2.G plasmids (gifts from Prof. Tieshan Tang’s lab at the Institute of Zoology, Chinese Academy of Sciences), using polyethylenimine (PEI) as the transfection reagent. The medium was refreshed 24 h post-transfection. After an additional 48 h, the conditioned medium was harvested and used to transduce MEG-01 cells with lentiviral particles carrying either pWPLXd-GFP-SLC35D3 or pWPLXd-SLC35D3-mCherry for subsequent fluorescent imaging.

### Immunofluorescence

HeLa, HEK293 or MEG-01 cells were seeded onto 14-mm glass coverslips and transfected with plasmids encoding either wild-type GFP-SLC35D3 or its variants for 24 h. The cells were either fixed directly followed by permeabilization with 0.2% Triton X-100 and subsequently immunostained with serotonin antibody (Sigma-Aldrich, S5545) to label DG precursors. HeLa cells were transfected with plasmids encoding either wild-type GFP-HA-SLC35D3 or its variants for 24 h. The cells were either fixed directly (control, non-permeabilized) or fixed followed by permeabilization with 0.05% Triton X-100 and subsequently immunostained with HA antibody (Cell Signaling Tech, 3724T) to visualize. Images were acquired using NIKON C2 confocal microscopy (Japan).

### Live-cell imaging

HeLa cells were cultured in 35-mm glass-bottomed dishes (Cellvis, D35-10-1-N) and transfected with either wild-type GFP-SLC35D3 or its variants for 24 h. The cells were subsequently labeled with CellMask Deep Red (ThermoFisher Scientific, C10046) to delineate the plasma membrane. Images were acquired using ZEISS LSM 880 (Germany) or NIKON C2 confocal microscopy.

### Line-scan analysis of protein localization

To assess the subcellular distribution of SLC35D3 variants, line-scan analysis was performed using ImageJ/Fiji (NIH). A straight line was drawn across individual cells, traversing both the plasma membrane and the intracellular compartment. Fluorescence intensity profiles were generated along each line, and the area under the curve at the peak of plasma membrane intensity (*A*_*PM*_) and the corresponding area under the curve of intracellular intensity (*A*_*intra*_) were measured. The intracellular-to-PM intensity peak area ratio (*A*_*intra*_*/A*_*PM*_) was calculated for each cell. Cells with *A*_*intra*_/*A*_*PM*_ > 80% were scored as PM-localized. For each experimental condition, at least 14 cells from three independent experiments were analyzed.

### Statistical analysis

Statistical significance was ascertained utilizing unpaired *t*-tests for pairwise comparisons, and one-way or two-way analysis of variance (ANOVA) for multiple group comparisons, and Chi-square (and Fisher’s exact) test with Bonferroni correction for pairwise multiple comparisons. Data from at least three experiments were presented as mean ± SD, as indicated in the figure legends. Differences were considered statistically significant at *p* < 0.05, with significance indicated in Figures as ∗*p* < 0.05, ∗∗*p* < 0.01, ∗∗∗*p* < 0.001. “ns” indicates no significant difference (*p* > 0.05). All statistical analyses were done using GraphPad Prism 8.0.

## Data availability

Materials, including raw data used for analysis and figure generation, plasmids, and cell lines generated in this study, are available from the corresponding author upon reasonable request.

## Supporting information

This article contains supporting information materials in Table S1–S5, and supporting data in Figure S1-S3.

## Conflict of interest

The authors declare that they have no conflicts of interest with the contents of this article.
