# Supplementary material for: Deletion of a YEDL sequence in SLC35D3 perturbs its targeting to platelet dense granule precursors
Source: J Biol Chem. 2026 Jul 16;302(9):113335. doi: 10.1016/j.jbc.2026.113335 (PMC13485648; doi:10.1016/j.jbc.2026.113335)
Supplement: Supplementary file 1 [file mmc1.docx]

**Supplemental to**

**Deletion of a YEDL sequence in SLC35D3 perturbs its targeting to platelet dense granule precursors**

Chentong Wang^1, #^, Yuanying Chen^1, #^, Wenyan Zhang^1^, Yefeng Yuan^1^, Qiaochu Wang^1,^ *, Chanjuan Hao^1, 2,^ *, and Wei Li^1, 2,^ *

**I. Supplementary Tables**

**Table S1. Antibodies information**

| **Antibody** | **Host Species** | **Source** |
| --- | --- | --- |
| anti-SLC35D3 | Rabbit Polyclonal | Thermo Fisher (PA572721) |
| anti-β-Actin | Mouse Monoclonal | Sigma-Aldrich (A5441) |
| anti-Flag | Mouse Monoclonal | Sigma-Aldrich (F1804-50UG) |
| anti-Flag M2 Agarose | Mouse Monoclonal | Sigma-Aldrich (A2220) |
| anti-AP1M1 | Rabbit Polyclonal | Proteintech (12112-1-AP) |
| anti-AP2M1 | Rabbit Polyclonal | Abcam (ab75995) |
| anti-AP3M1 | Rabbit Polyclonal | Abcam (ab201227) |
| anti-GFP | Rabbit Polyclonal | Easybio (BE2002) |
| anti-Dysbindin | Rabbit Polyclonal | Cell Signaling Tech (4611S) |
| anti-HA | Mouse Monoclonal | Cell Signaling Tech (3724T) |
| anti-Serotonin | Rabbit Polyclonal | Sigma-Aldrich (S5545) |

**Table S2. Reagents information**

| **Reagent** | **Source** | **Purpose** |
| --- | --- | --- |
| Lipofectamine 3000 | Thermo Fisher (L3000015) | Transfection |
| Polyethylenimine Linear | Yeasen (40816ES03) | Transfection |
| Lipofectamine RNAiMAX | Thermo Fisher (13778100) | Transfection |
| Cycloheximide | MedChemExpress (HY-12320) | Protein synthesis inhibitor |
| MG-132 | MedChemExpress (HY-13259) | Proteasome inhibitor |
| Bafilomycin A1 | Sigma-Aldrich (88899-55-2) | Lysosomal inhibitor |
| Mepacrine | Sigma-Aldrich (Q2876) | Dense granule marker |
| CellMask Deep Red | Thermo Fisher (C10046) | Plasma membrane labeling |
| KOD-Plus-Neo kit | TOYOBO (KOD-401) | Construction of plasmid mutations |
| Brilliant Blue G | Beyotime (ST1119) | Protein band detection |

**Table S3. Plasmids information**

| **Plasmid Name** | **Description** | **Source/Construction** |
| --- | --- | --- |
| pWPLXd-TMCO1 | Plasmid backbone | A gift from Prof. Tieshan Tang’s lab (Institute of Zoology, CAS) |
| pWPLXd-GFP-SLC35D3 | N-terminal GFP-tagged full-length SLC35D3 | Constructed by replacing TMCO1 in pWPLXd-TMCO1 with GFP-SLC35D3 |
| pWPLXd-GFP-SLC35D3 ΔY^311^EDL | GFP-SLC35D3 with Y^311^EDL motif deleted | Site-directed mutagenesis |
| pWPLXd-SLC35D3-mCherry | C-terminal mCherry-tagged full-length SLC35D3 | Constructed by replacing TMCO1 in pWPLXd-TMCO1 with SLC35D3-mCherry |
| GFP-SLC35D3 | N-terminal GFP-tagged full-length SLC35D3 | Our Lab |
| Flag-SLC35D3 | N-terminal Flag-tagged full-length SLC35D3 | Our Lab |
| Flag-Dysbindin | N-terminal Flag-tagged full-length Dysbindin | Our Lab |
| LAMP1-GFP | C-terminal GFP-tagged full-length LAMP1 | Our Lab |
| Rab5a-GFP | C-terminal GFP-tagged full-length Rab5a | Our Lab |
| TPC1-mCherry | Plasmid backbone | A gift from Antony Galione |
| mRuby2-GS28 | N-terminal mRuby2-tagged full-length GS28 | A gift from Prof. Liang Ge’s lab (School of Life Sciences, Tsinghua University) |
| SLC35D3-mCherry | C-terminal mCherry-tagged full-length SLC35D3 | Constructed by replacing TPC1 in TPC1-mCherry with SLC35D3 |
| GFP-SLC35D3 ΔY^311^EDL | GFP-SLC35D3 with Y^311^EDL motif deleted | Site-directed mutagenesis |
| GFP-SLC35D3 ΔY^389^LEV | GFP-SLC35D3 with Y^389^LEV motif deleted | Site-directed mutagenesis |
| GFP-SLC35D3 ΔY^311-CT^ | GFP-SLC35D3 with C-terminal truncation removing aa 311–416 | Site-directed mutagenesis |
| GFP-SLC35D3 ΔY^389-CT^ | GFP-SLC35D3 with C-terminal truncation removing aa 389–416 | Site-directed mutagenesis |
| GFP-SLC35D3 ΔR^394-CT^ | GFP-SLC35D3 with C-terminal truncation removing aa 394–416 | Site-directed mutagenesis |
| GFP-SLC35D3 Y^311^EDL/AAAA | The Y^311^EDL of GFP-SLC35D3 is replaced by four alanines | Site-directed mutagenesis |
| GFP-SLC35D3 Y^389^LEV/AAAA | The Y^389^LEV of GFP-SLC35D3 is replaced by four alanines | Site-directed mutagenesis |
| GFP-SLC35D3 Y^311^/A | The Y^311^ of GFP-SLC35D3 is replaced by alanine | Site-directed mutagenesis |
| GFP-SLC35D3 Y^311^/F | The Y^311^ of GFP-SLC35D3 is replaced by phenylalanine | Site-directed mutagenesis |
| GFP-HA-SLC35D3 | The HA tag is cloned in GFP-SLC35D3 | Constructed by inserting HA tag after the 277th aa residue of SLC35D3 in GFP-SLC35D3 |
| GFP-HA-SLC35D3 ΔY^311^EDL | The HA tag is cloned in GFP-SLC35D3 ΔY^311^EDL | Constructed by inserting HA tag after the 277th aa residue of SLC35D3 in GFP-SLC35D3 ΔY^311^EDL |
| GFP-HA-SLC35D3 Y^311^EDL/AAAA | The HA tag is cloned in GFP-SLC35D3 Y^311^EDL/AAAA | Constructed by inserting HA tag after the 277th aa residue of SLC35D3 in GFP-SLC35D3 Y^311^EDL/AAAA |
| Flag-SLC35D3 ΔY^311^EDL | Flag-SLC35D3 with Y^311^EDL motif deleted | Site-directed mutagenesis |
| pGEX-4T-1 | Plasmid backbone | Our Lab |
| GST-SLC35D3 311-416 | GST-SLC35D3 with C-terminal fragment including aa 311-416 | Constructed by inserting SLC35D3 with C-terminal fragment including aa 311-416 in pGEX-4T-1 |
| GST-SLC35D3 315-416 | GST-SLC35D3 with C-terminal fragment including aa 315-416 | Constructed by inserting SLC35D3 with C-terminal fragment including aa 315-416 in pGEX-4T-1 |
| Flag-AP1M1 | Expression vector for Flag-tagged μ1 subunit (AP1M1) of AP-1 | Cloned in-house from HeLa cDNA |
| Flag-AP2M1 | Expression vector for Flag-tagged μ2 subunit (AP2M1) of AP-2 | Cloned in-house from HeLa cDNA |
| Flag-AP3M1 | Expression vector for Flag-tagged μ3A subunit (AP3M1) of AP-3 | Cloned in-house from HeLa cDNA |
| psPAX2 | Lentivirus envelope plasmid | A gift from Prof. Tieshan Tang’s lab (Institute of Zoology, CAS) |
| pMD2.G | Lentivirus envelope plasmid | A gift from Prof. Tieshan Tang’s lab (Institute of Zoology, CAS) |

**Table S4. siRNA primer sequences information**

| **siRNA** | **Sequences (5’→3’)** |
| --- | --- |
| AP1M1 HOMO-1 | Forward 5’-AAAGUACUCGGAGAAGACC-3’  Reverse 5’-GGUCUUCUCCGAGUACUUU-3’ |
| AP1M1 HOMO-2 | Forward 5’-AACUGCCUCAAUGACGUCC-3’  Reverse 5’-GGACGUCAUUGAGGCAGUU-3’ |
| AP1M1 HOMO-3 | Forward 5’-AUGGACCACACGAUCUCGGC-3’  Reverse 5’-GCCGAGAUCGUGUGGUCCAU-3’ |
| AP1M1 HOMO-4 | Forward 5’-UCCGAAGGCAUCAAGUAUCGGAAGA-3’  Reverse 5’-UCUUCCGAUACUUGAUGCCUUCGGA-3’ |
| AP2A1 HOMO-1 | Forward 5’-GAGCAUGUGCACGCUGGCCA-3’  Reverse 5’-UGGCCAGCGUGCACAUGCUC-3’ |
| AP2M1 HOMO-2 | Forward 5’-AGUGGAUGCCUUUCGGGUCAUU-3’  Reverse 5’-AAUGACCCGAAAGGCAUCCACU-3’ |
| AP3M1 HOMO-1 | Forward 5’-GGAAUUACAGUGACAGUUC-3’  Reverse 5’-GAACUGUCACUGUAAUUCC-3’ |
| AP3M1 HOMO-2 | Forward 5’-GGUAGUAAAAGGGAACAUU-3’  Reverse 5’-AAUGUUCCCUUUUACUACC-3’ |
| AP3D1 HOMO-1 | Forward 5’-GCGAGAAACUGCCUAUUCA-3’  Reverse 5’-UGAAUAGGCAGUUUCUCGC-3’ |
| AP3D1 HOMO-2 | Forward 5’-UCUGCAAGCUGACGUAUUU-3’  Reverse 5’-AAAUACGUCAGCUUGCAGA-3’ |
| Dysbindin HOMO-1 | Forward 5’-AAGUGACAAGUCAAGAGAAGCAA-3’  Reverse 5’-UUGCUUCUCUUGACUUGUCACUU-3’ |
| Dysbindin HOMO-2 | Forward 5’-UGGAUUAGAAUUACUUAGC-3’  Reverse 5’-GCUAAGUAAUUCUAAUCCA-3’ |

**Table S5. Software and computational tools**

| **Tool** | **Source** | **Purpose** |
| --- | --- | --- |
| Eukaryotic Linear Motif | http://elm.eu.org/ | Prediction of protein sorting motifs |
| DeepTMHMM | https://dtu.biolib.com/DeepTMHMM | Transmembrane topology prediction and classification |
| Clustal Omega | https://www.ebi.ac.uk/Tools/msa/clustalo/ | Multiple sequence alignment of protein homologs |
| Image J | NIH | Image analysis |
| GraphPad Prism 8.0 | GraphPad Software | Statistical analysis and graphing |
| ZEISS LSM 880 & NIKON C2 | ZEISS / NIKON | Confocal laser scanning microscopy |

**II. Supplementary Figures and Figure Legends**

**Figure S1.** **Secondary structure and evolutionary conservation analysis of SLC35D3 and potential AP binding motifs of SLC35D3.** *A* and *B,* The predicted structure of SLC35D3 featuring transmembrane (TM) helices was analyzed by DeepTMHMM. The lengths of the boxes correspond to the number of amino acids they contain, with their relative positions indicated. Potential AP binding sequences, including two potential tyrosine-based signals (Y^311^EDL and Y^389^LEV) and two potential di-leucine-based motifs (RGVPL^372^V and EVWRL^395^V) were plotted to the C-terminus of human SLC35D3. The RRLGL^64^I motif, predicted to be located within TM3, has been excluded from consideration as an AP binding motif. ELM: Eukaryotic Linear Motif. *C,* The alignment of amino acid sequences of SLC35D3 homologs reveals the conservation of specific motifs across species, including Y^311^EDL, RGVPL^372^V, Y^389^LEV, and EVWRL^395^V, which are hypothesized to function as adaptor protein (AP) binding motifs. The black solid-line squares denote di-leucine-based sorting motifs, whereas the black dashed-line squares indicate tyrosine-based sorting signals. Note that the RGVPL^372^V motif are not conserved across species. *D,* Quantification of the percentage of SLC35D3 localized to the plasma membrane (PM) and non-plasma membrane (Non-PM) fractions according to the line‑scan analysis. The percentage of ΔY^311-CT^ cells on PM (83.3%, n = 30) was significantly higher than that of WT cells (0%, n = 77), *p* < 0.001. Similarly, ΔY^389-CT^ cells showed a significantly higher proportion (78.8%, n = 33) compared to WT, *p* < 0.001. No significant difference was observed between WT and ΔR^394-CT^, Y^311^EDL/AAAA, or Y^389^LEV/AAAA mutants. *E,* The percentage of ΔY^311^EDL mutant cells on PM (69.4%, n = 36) was significantly higher than that of WT cells (0%, n = 77), *p* < 0.001. No significant difference was observed between WT and ΔY^389^LEV mutant. Statistical significance was determined by Chi-square (and fisher’s exact) test with Bonferroni correction for pairwise multiple comparisons, ns, *p* > 0.05, ****p* < 0.001.

**Figure S2. SLC35D3 mutants ΔY^389^LEV, ΔY^311-CT^, and ΔY^389-CT^ exhibit significantly increased Golgi localization.** *A,* Schematic depiction of the SLC35D3 topology structure of full-length and the ΔY^311^EDL deletion. In vesicle membranes, the N-terminus and C-terminus of SLC35D3 are located on the cytoplasmic side. After introducing an HA tag into the C-terminal luminal side region (residues 274-280), the HA tag cannot be recognized by the HA antibody in the absence of Triton X-100 permeabilization, confirming that this region is in the intracellular environment. When the YEDL motif at the C-terminus is deleted (ΔY^311^EDL), SLC35D3 ΔY^311^EDL undergoes ectopic localization to the plasma membrane (PM), accompanied by topological inversion, with both the N-terminus and C-terminus facing the cytoplasmic side. At this time, the HA tag is exposed to the extracellular environment and can be recognized by the HA antibody, verifying the PM location of SLC35D3 ΔY^311^EDL. *B,* Quantification of the percentage of SLC35D3 localized to the plasma membrane (PM) and non-plasma membrane (Non-PM) fractions under non‑permeabilized conditions. Quantification analysis showed that the percentage of cells on PM in ΔY^311^EDL mutant (89.3%, n = 28) is significantly higher than that in WT (0%, n = 26), *p <* 0.001. Statistical significance was determined by Chi-square (and fisher’s exact) test with Bonferroni correction for pairwise multiple comparisons, ns, *p* > 0.05, ****p* < 0.001. *C,* Confocal fluorescence images of GFP-SLC35D3 (pseudo color green) and mRuby2-GS28 (Golgi apparatus marker) (pseudo color magenta) in HeLa cells. Representative images show WT SLC35D3 (GFP-SLC35D3), ΔY^389^LEV, ΔY^311-CT^, and ΔY^389-CT^ mutants. Compared with WT SLC35D3, the ΔY^389^LEV deletion, ΔY^311-CT^ and ΔY^389-CT^ truncation mutants of SLC35D3 showed significantly more pronounced localization at the perinuclear Golgi region. The enlarged pictures showed magnified images of the boxed region. Scale bars represent 10 μm for overview images and 2 μm for magnified sections. *D,* Quantification of colocalization was performed using Manders’ coefficient M1, which quantifies the fraction of SLC35D3 area in GS28 area. (ΔY^389^LEV: 0.55 ± 0.08, n = 43; ΔY^311-CT^: 0.62 ± 0.18, n = 89; ΔY^389-CT^: 0.49 ± 0.18, n = 53, vs. WT: 0.22 ± 0.13, n = 84; *p* < 0.001). *E,* Co-IP assays were performed to ascertain the association between overexpressed GFP-SLC35D3, GFP-SLC35D3 ΔY^311^EDL, the alanine substitution variant of Y311 (Y^311^/A), the phenylalanine substitution variant of Y311 (Y^311^/F), and the complete alanine substitution variant of Y^311^EDL (Y^311^EDL/AAAA) with the AP-3 complex (Flag-AP3M1). The star sign (*) represents endogenous AP3M1. The results indicated that the absence of the Y^311^EDL motif in SLC35D3 weakened the binding affinity for Flag-AP3M1. In contrast, other mutants did not alter binding to the AP-3 complex. *F,* Quantification of relative AP3M1 protein levels pulled down by the wild-type (WT) versus the Y^311^EDL motif deletion mutant (ΔY^311^EDL), two single-residue substitution mutants at the critical tyrosine position (Y^311^/A and Y^311^/F), and the full alanine-substitution variant (Y^311^EDL/AAAA) constructs demonstrated a statistically significant reduction in AP3M1 binding by the ΔY^311^EDL construct compared to WT (0.40 ± 0.09, n = 3, *p* < 0.01), confirming that deletion of the Y^311^EDL motif consistently impaired binding to AP3M1. Data were presented as mean ± SD. Statistical significance was assessed by one-way analysis of variance (ANOVA) for multiple group comparisons, ns, *p* > 0.05, ***p* < 0.01, ****p* < 0.001.

**Figure S3. SLC35D3 translocates to the PM in AP-2 and AP-3 knockdown cells.** *A-D,* Live-cell imaging was employed to ascertain the PM localization of SLC35D3 following knockdown of AP-1 (siAP-1) (B), AP-2 (siAP-2) (C) or AP-3 (siAP-3) (D). SLC35D3 was predominantly localized to the PM (C and D) compared with the control (siNC) (A). HeLa cells overexpressing GFP-SLC35D3 (pseudo color green) were subsequently stained with Cell Mask (pseudo color magenta), a fluorescent dye that binds to the plasma membrane. The white dashed lines delineate the cellular regions within the merged panels where the fluorescence intensities for the green and magenta channels were quantified. Fluorescence intensities were depicted using the plot profile method in Image J software as shown in the right panel. The scale bars corresponded to 10 μm for the overview images and 2 μm for the magnified sections. *E,* Quantitative analysis of the fluorescence intensity of SLC35D3 on the PM after knockdown of AP-1 (siAP-1: 6015 ± 1974, n = 32, vs. siNC: 4587 ± 1597, n = 36, *p* = 0.2217), AP-2 (siAP-2: 11607 ± 6315, n = 26, vs. siNC: 4587 ± 1597, n = 36, *p* < 0.001), AP-3 (siAP-3: 6674 ± 2339, n = 25, vs. siNC: 4587 ± 1597, n = 36, *p* = 0.0374). Statistical significance was assessed by one-way analysis of variance (ANOVA) for multiple group comparisons, ns, *p* > 0.05, **p* < 0.05, ****p* < 0.001. *F-H,* Representative protein expression levels of the AP1M1, AP2M1 and AP3M1 were assessed to evaluate the knockdown efficiencies. β-Actin was used as a loading control. HeLa cells transfected with siRNAs for 72 hours were lysed for Western blotting. The intensity of the bands was quantified using Image J software and normalized relative to the corresponding β-actin bands. Quantitative analysis of knockdown efficiency (54.6% decrease at siAP-1, n = 3, vs. siNC, 0.45 ± 0.06, n = 3; 69.1% decrease at siAP-2, n = 4, vs. siNC, 0.31 ± 0.14, n = 3; 61.7% decrease at siAP-3, n = 7, vs. siNC, 0.38 ± 0.14, n = 3). The siNC values were normalized to a baseline of 1. Data are presented as mean ± SD. Statistical significance was assessed by two-tailed unpaired *t*-test. ****p* < 0.001. *I,* The assessment of AP-3 knockdown efficiency was based on measuring AP3M1 subunit protein levels within the AP-3 complex, with β-actin utilized as a reference for normalization. Quantitative analysis of AP-3 knockdown efficiency revealed statistically significant differences at each time point. (0 h: siAP-3: 0.54 ± 0.10, ****p* < 0.001; 3 h: siAP-3: 0.41 ± 0.10, ****p* < 0.001; 6h: siAP-3: 0.60 ± 0.12, ****p* < 0.001; 9 h: siAP-3: 0.42 ± 0.06, ****p* < 0.001; 12h: siAP-3: 0.46 ± 0.04, ****p* < 0.001). *J,* The assessment of BLOC-1 knockdown efficiency was based on measuring Dysbindin subunit protein levels, with β-actin utilized as a reference for normalization. For BLOC-1 knockdown, significantly reduced Dysbindin levels were observed at each time point. (0 h: siBLOC-1: 0.57 ± 0.06, ****p* < 0.001; 3 h: siBLOC-1: 0.62 ± 0.08, ****p* < 0.001; 6h: siBLOC-1: 0.47 ± 0.13, ****p* < 0.001; 9 h: siBLOC-1: 0.44 ± 0.17, ****p* < 0.001; 12h: siBLOC-1: 0.49 ± 0.11, ****p* < 0.001). HEK293 cells transfected with siRNAs for 72 hours were lysed for Western blotting. The intensity of the bands was quantified using Image J software and normalized to the corresponding β-actin bands. The siNC values were normalized to a baseline of 1. Data are presented as mean ± SD, n = 3 if not specified. Statistical significance was assessed by two-tailed unpaired *t*-test.
